# Insights into the human metabolism and in silico receptor activity of gidazepam and desalkylgidazepam

**DOI:** 10.1007/s00204-025-04249-z

**Published:** 2025-12-08

**Authors:** Prince Sellase Gameli, Johannes Kutzler, Cristina Minnelli, Giuseppe Basile, Emiliano Laudadio, Francesco Paolo Busardò, Volker Auwärter, Jeremy Carlier

**Affiliations:** 1 Department of Biomedical Sciences and Public Health, Marche Polytechnic University, Via Tronto 10/a, 60126 Ancona, Italy; 2 Institute of Forensic Medicine, Medical Center, University of Freiburg, Freiburg, Germany; 3 Medical Faculty, University of Freiburg, Freiburg, Germany; 4 Department of Life and Environmental Sciences, Marche Polytechnic University, Ancona, Italy; 5 Department of Science and Engineering of Matter, Environment and Urban Planning, Marche Polytechnic University, Via Brecce Bianche 12, 60131 Ancona, Italy

**Keywords:** Metabolism, Consumption biomarker, High-resolution mass spectrometry, Molecular dynamics, GABA_A_ receptor, TSPO

## Abstract

Desalkylgidazepam, an active gidazepam metabolite, first appeared on the illicit drug market in 2022 and has been detected in polydrug intoxication cases. Since both benzodiazepines and their metabolites can result from gidazepam metabolism, it is important to identify markers that specifically indicate consumption of each compound. We therefore investigated the human metabolism of gidazepam and desalkylgidazepam by incubating them with human hepatocytes and analyzing the resulting samples, along with human blood from a confirmed desalkylgidazepam-positive case, using liquid chromatography-high-resolution mass spectrometry. To further assess their pharmacological profile, we examined the activity of gidazepam, desalkylgidazepam, and their potential (3*R*)- and (3*S*)-hydroxy metabolites at γ-aminobutyric acid A (GABA_A_R) and 18 kDa translocator protein (TSPO) receptors in silico, using AutoDock Tools and UCSF Chimera. Gidazepam was metabolized through *N*-desalkylation (yielding desalkylgidazepam), *N*-acetylation, and *N*-glucuronidation. Conversely, desalkylgidazepam was subjected to hydroxylation and subsequent *O*-glucuronidation reactions. Notably, gidazepam demonstrated a lower affinity at GABA_A_R’s prominent α_1_/γ_2_ site compared to desalkylgidazepam and its (3*R*)- and (3*S*)-hydroxy metabolites. However, its interaction with the transmembrane domains of the α_1_β_2_ subunit may account for its anxiolytic effects. For the TSPO receptor, gidazepam and 3-hydroxy desalkylgidazepam metabolites showed higher binding affinity, whereas desalkylgidazepam did not bind to TSPO. Our findings suggest blood markers specific to gidazepam, namely gidazepam-*N*-glucuronide and *N*-acetyl gidazepam, are essential for confirming gidazepam consumption. In addition, in silico modelling supports the hypothesis that gidazepam functions as a prodrug via GABA_A_R and as an agonist at TSPO. Further research is necessary to clarify designer benzodiazepine activity at TSPO.

**Supplementary Information:**

The online version contains supplementary material available at 10.1007/s00204-025-04249-z.

## Introduction

New psychoactive substance (NPS) use poses a growing public health concern worldwide as evidenced by an ongoing increase in drug seizures, intoxication cases, and fatalities involving NPS (Greenblatt and Greenblatt 2019; United Nations Office on Drugs and Crime 2025a). Designer benzodiazepines, a subset of NPSs including substances not approved for medical use (e.g., bretazenil), regionally prescribed pharmaceuticals (e.g., etizolam), and active metabolites (e.g., fonazepam), have emerged as prominent contributors to this trend (Moosmann et al., 2015; El Balkhi and Abbara 2023). Currently, over 30 designer benzodiazepines are monitored by international agencies tasked with surveilling their emergence.

Gidazepam was first developed in 1997 and marketed as an anxiolytic with legal status limited to Russia and Ukraine (InterChem 2025). Reports of its detection outside these countries are rare; however, in 2022 the drug was seized for the first time in New Zealand (United Nations Office on Drugs and Crime 2025b). Gidazepam is metabolized via *N*-desalkylation to desalkylgidazepam, an active metabolite, frequently detected in forensic toxicology casework. Major urinary metabolites, carboxymethylgidazepam, and minor ones such as 3-hydroxydesalkylgidazepam glucuronide have been observed, although no blood markers were reported (Maskell et al., 2023).

Desalkylgidazepam appeared on the NPS markets in the US, and Canada in 2022, and subsequently in several European and Asian countries, including China and Singapore (Krotulski et al., 2022; Merette et al., 2023; United Nations Office on Drugs and Crime 2025c). As of 2024, it was the second most prevalent designer benzodiazepine in the US after bromazolam (Krotulski et al., 2025). In a recent Canadian study, desalkylgidazepam was identified in 63 postmortem cases, often in combination with opioids and other drugs, highlighting its key role in fatal polydrug intoxications (Merette et al. 2023).

Pharmacologically, benzodiazepines, including gidazepam and desalkylgidazepam, act as positive allosteric modulators of γ-amino butyric acid (GABA) at the GABA_A_ receptor (GABA_A_R), the main inhibitory neurotransmitter receptor in the central nervous system (Zhu et al., 2022; Gameli et al., 2024). GABA_A_R is a complex ionotropic pentamer involved in inhibitory neurotransmission leading to sedating, anxiolyzing, muscle relaxing, and sleep-inducing effects (Ghit et al., 2021). Presently, nineteen GABA_A_R subunits are reported, however, the most prevalent combination is the α_1_β_2_γ_2_ complex. (Goetz et al., 2007). In vitro studies, suggest gidazepam is a GABA_A_R partial agonist, whereas desalkylgidazepam or the 3-hydroxy desalkylgidazepam metabolites demonstrate distinct activity profiles (Maskell et al. 2023).The molecular mechanism underlaying this difference, however, remains unexplored. Furthermore, gidazepam’s relatively high affinity for the translocator protein (TSPO 18 kDa), previously the peripheral benzodiazepine receptor, is reportedly three times greater than at GABA_A_R (Korkhov et al., 2002; Maskell et al., 2023). The pharmacodynamic consequences of this interaction, potentially involving modulation of neurosteroid synthesis, are also not fully understood (Wolf et al., 2015; Cheung et al., 2023).

The rapid conversion of gidazepam to desalkylgidazepam poses challenges for analytical identification, and in interpretating toxicological findings. Consequently, it is crucial to investigate their biotransformational pathways and identify specific analytical markers capable of distinguishing gidazepam and desalkylgidazepam intake. In this study, we characterized gidazepam and desalkylgidazepam human metabolism using donor pooled human hepatocytes and blood from an authentic intoxication case via liquid chromatography-high-resolution mass spectrometry (LC-HRMS), and employed in silico molecular modelling to investigate their interaction at GABA_A_R and TSPO 18 kDa receptor.

## Materials and method

### Chemicals and materials

Pure standards of gidazepam and desalkylgidazepam were provided within the EU-ADEBAR plus project (Pulver et al. 2022), and diclofenac was obtained from Sigma Aldrich (Milan, Italy). Methanolic stock solutions at 1 mg/mL were prepared and stored at − 20 °C. LC–MS grade acetonitrile, water, methanol, and formic acid were purchased from Carlo Erba (Cornaredo, Italy). Human hepatocytes pooled from ten donors, thawing medium, and Trypan blue (0.4%) were obtained from Lonza (Basel, Switzerland). Williams’ medium E, HEPES buffer (2-[4-(2-hydroxyethyl)-1-piperazinyl] ethanesulfonic acid), and *L*-glutamine were purchased from Sigma Aldrich. HEPES and *L*-glutamine, 2 and 20 mmol/L, respectively, were used in preparing Supplemented William’s medium E (SWM) and stored at  +  4 °C until incubations.

### *In silico* metabolites prediction

Gidazepam and desalkylgidazepam metabolites were predicted with GLORYx, a free web-based prediction tool, by inserting Simplified Molecular-Input Line-Entry System (SMILES) generated using ACD/ChemSketch (freeware) in the “*phase I and II metabolism*” option (Stork et al., 2020; De Bruyn Kops et al., 2021). Predicted first- and second-generation metabolites with scores ≥  0.20 were considered and added as an inclusion list (see Table S1) for HRMS/MS acquisition.

### Incubation with human hepatocytes

Gidazepam and desalkylgidazepam were incubated separately with hepatocytes using a protocol previously described (Carlier et al., 2018), with minor modifications. Briefly, hepatocytes were thawed in 50 mL thawing medium (TM), then centrifuged for 5 min at 50–100 g*.* The resulting pellets were resuspended in 50 mL SWM, and after a second centrifugation, the supernatant was discarded. The pellets were then resuspended in 2 mL SWM. Cell viability was determined using the Trypan exclusion method. The volume of SWM was then adjusted to yield 2 × 10^6^ viable cells/mL. Subsequently, 250 µL of hepatocyte suspension and 250 µL of either gidazepam or desalkylgidazepam (20 µmol/L in SWM) were gently combined in a sterile 24-well plate. The mixture was incubated for three hours using an ArgoLab incubator (Arezzo, Italy). To monitor the experiment, positive (diclofenac) and negative controls were included under the same experimental conditions. The reaction was terminated by adding 500 μL ice-cold acetonitrile, followed by centrifugation at 15,000 g for 10 min. The samples were then stored at − 80 °C until further analysis.

### Authentic blood specimen and sample preparation

Desalkylgidazepam, quantified at 620 ng/mL, and gidazepam were found in authentic blood in an intoxication casework. Quantification was achieved using liquid chromatography-tandem mass spectrometry analysis using a validated method accredited under ISO/IEC 17025 for forensic analysis. Samples were analyzed following an updated and revalidated version of the previously published method for designer benzodiazepines (Moosmann et al., 2014; Koch et al., 2018).

For LC-HRMS analysis, both hepatocyte incubates and the blood specimen were thawed at room temperature, centrifuged for 10 min at 15,000 g*,* and mixed with acetonitrile in 1:1 ratio for hepatocyte incubates, and 1:2 for blood. The mixtures were centrifuged again at 15,000 g for 10 min and dried under nitrogen at 37 °C. The resulting precipitate was reconstituted with mobile phase A: mobile phase B (90:10 *v/v,* see UHPLC Section), spun under the same conditions, and 10 µL was injected for analysis.

### Instrumental conditions

The LC-HRMS system, used for analyzing hepatocyte incubates and authentic blood sample, consisted of a DIONEX Ultimate 3000 system paired with a Q-Exactive quadrupole orbitrap hybrid high-resolution mass spectrometer, equipped with a heated-electrospray ionization (HESI) source from Thermo Scientific (Waltham, MA, USA). HRMS data were acquired in both positive- and negative-ionization modes for comprehensive analysis.

### Ultrahigh-performance liquid chromatography

Chromatographic separation was achieved using a Kinetex® Biphenyl column (150 × 2.1 mm, 2 μm; Phenomenex, Torrance, CA, USA), maintained at 37 °C throughout the run. The mobile phases consisted of 0.1% formic acid in water (mobile phase A) and 0.1% formic acid in acetonitrile (mobile phase B). Gradient elution began with 98:2 (mobile phase A: B) for the first 2 min, B was gradually increased to 45% over the course of 15.5 min, and then quickly raised to 95% within 1 min. This elution was held until 19.5 min, after which the starting conditions were reinstated within 0.1 min and maintained until 24.0 min. The flow rate was kept constant at 0.4 mL/min throughout the run.

### High-resolution mass spectrometry

The HESI source conditions were optimized using 1 μg/mL of gidazepam and desalkylgidazepam standards dissolved in mobile phases A:B, 90:10 (*v/v*). The following source parameters were applied: spray voltage, ± 3.5 kV; capillary and auxiliary temperature, 300 °C; sheath gas, 5 arbitrary units (AU); auxiliary gas, 50 AU; and S-lens frequency at 50.

Data acquisition utilized both full-scan mass spectrometry (MS) and data-dependent tandem mass spectrometry (ddMS/MS) modes. For full-scan MS, settings were: automatic gain control target, 3 × 10^6^; resolution, 70,000 (at *m/z* 200, full width at half maximum); maximum injection time, 256 ms; and scan range, *m/z* 250–700. The settings for ddMS/MS acquisition were as follows: automatic gain control target, 2 × 10^5^; minimum target, 6.5 × 10^2^; resolution, 17,500; maximum injection time, 64 ms; and isolation window, *m/z* 1.2. The normalized collision energies were set at 20, 40, and 70 AU, using five loops; with dynamic exclusion at 2.0 s.

To ensure better accuracy, HRMS calibration was performed prior to analysis using a lock mass list. Additionally, an inclusion list for ^79^Br and ^81^Br in both positive- and negative-ionization modes were employed, to ensure the acquisition of un-interfered ddMS/MS spectra resulting from possible isotopic distribution of gidazepam and desalkylgidazepam. The inclusion list was created based on reported biotransformations from the literature and in silico metabolite predictions using GLORYx.

### Metabolites identification

HRMS data was processed with Compound Discoverer v3.1.1.12 (Thermo Scientific, Waltham, MA, USA) with a semi-automated workflow. This workflow combined both targeted and non-targeted approaches, adapting a previously published method (Di Trana et al., 2021). Briefly, ions detected in HRMS/MS were compared with theoretically generated metabolites, using an intensity threshold of 5 × 10^3^ and a 5 ppm mass tolerance. Subsequently, both the MS/MS spectra and the proposed elemental compositions of predicted and unpredicted metabolites were compared against databases available in mzCloud (including counterfeit drugs, drugs of abuse/illegal drugs, illegal additives, and therapeutics/prescription drugs databases) and ChemSpider (Cayman Chemical and DrugBank databases). For these comparisons, an intensity threshold of 10^5^ and mass tolerances of 5 and 10 ppm, respectively, were applied. Table S2 details the workflow, potential reactions, and predicted metabolites in HRMS software-aided data processing.

### *In silico* GABAAR and TSPO 18 kDa receptor activity

Molecular docking and dynamics simulations of gidazepam, desalkylgidazepam, and the potential (3*R*)-, and (3*S*)-hydroxy-desalkylgidazepam metabolites were conducted using AutoDock Suite 4.2 and AutoDock Tools. For comparative purposes, modelling studies of diazepam and 4-chlorodiazepam (Ro5-4864) were also performed. The three-dimensional structures of GABA_A_R and TSPO 18 kDa were obtained from 6 × 3z (Kim et al.,^2020^) and 8e7z (Liu et al., 2023), crystallographic or pdb files, respectively.

### Molecular docking studies

Each subunit of the GABA_A_R pentameric unit was treated separately and screened to identify possible binding poses. Docking calculations were performed using the AutoDock Suite 4.2 (Morris et al., 2009). Polar hydrogen atoms and partial charges were added to the receptor using Autodock tools (Sanner et al., 1999), while ligand charges were assigned with the AM1-BCC semi-empirical method in UCSF Chimera (Jakalian et al., 2002). Additionally, atomic solvation parameters and fragmental volumes for the proteins were assigned using the Addsol tool included in the software package. Flexible torsions in the ligands were specified with the Autotors module, permitting all dihedral angles to rotate freely. Affinity grid fields were generated with the auxiliary program Autogrid. For each GABA_A_R chain, an extended grid of 126 Å^3^ was used. The resulting docked conformations were then clustered into families of similar binding modes, using a root mean square deviation (RMSD) clustering tolerance of 2 Å. The conformations with the lowest docking energy in the most populated clusters were considered the most prominent binding site.

Subsequently, the GABA_A_R pentameric unit was reassembled, and all potential binding sites identified from the initial screening were re-analyzed using a more focused, “targeted” docking approach. In this step, each binding pocket was refined using a grid field of 44 Å^3^, and again, the lowest energy conformations from the most populated clusters were selected as the most stable orientations. Docking energy was defined as the sum of the intermolecular energy and the internal energy of each ligand. Free binding energy, conversely, was the sum of the intermolecular energy and the torsional free energy. The docking results were calculated using an empirical free energy force field, employing the Lamarckian genetic algorithm for rapid prediction of conformation and free energy. Notably, the calculated free binding energy is related to the inhibition constant K_i_ by the known thermodynamic relation ∆G = –RT ln K_i_.

For the TSPO 18 kDa protein, molecular docking was more straightforward compared to the GABA_A_R, as TSPO is monomeric and extremely small, with only its carboxy-terminal region external to the cell membrane. A grid of 44 × 42 × 40 Å^3^ was thus used for docking analysis, and the most stable ligand-receptor complexes were selected as starting points for subsequent molecular dynamics (MD) simulations.

### Molecular dynamics simulations

The receptor-ligand complexes obtained from molecular docking were subjected to 200 ns of MD simulations, following a series of minimization and equilibration steps with the AMBER99SB-ildn force field (Lindorff-Larsen et al., 2010). A 298 K temperature was used during minimization and equilibration step, while 310 K was utilized for the MD simulations. Both GABA_A_R and TSPO 18 kDa were oriented within a lipid environment containing 1-palmitoyl-2-oleoyl-glycero-3-phosphocholine (POPC), using the Orienting Protein Membrane server (Lomize et al., 2022).

For the GABA_A_R, a lipid system of 388 POPC molecules was constructed within a simulation box measuring 12.666 × 12.666 × 15.803 nm, containing 52,301 transferable intermolecular potential with three points (TIP3P) water molecules, as well as 142 Na^+^ and 164 Cl^−^ ions. On the other hand, the TSPO 18 kDa system comprised 140 POPC lipids, 9978 TIP3P water molecules, 26 Na^+^, and 27 Cl^−^ ions, within a simulation box of 7.454 × 7.454 × 10.034 nm.

Considering TSPO 18 kDa functions as a mitochondrial benzodiazepine receptor, an additional membrane was modelled using 138 1,1’-palmitoyl-2,2’-vacenoyl-cardiolipin (PVCL2) molecules, together with 17,681 TIP3P water molecules, 321 Na^+^, and 47 Cl^−^ ions within a simulation box of 9.874 × 9.874 × 10.034 nm. This setup allowed for comprehensive investigation of gidazepam, its active bromo-nordiazepam metabolite, and the 3-hydroxy metabolites of bromo-nordiazepam acting on the TSPO 18 kDa.

## Results

### *In silico* metabolites prediction

In total, eighteen metabolites were predicted using the web-based tool GLORYx. Of these, nine were first-generation metabolites (gidazepam, pM1 – pM6 and desalkylgidazepam, pM1 – pM3), and the remainder were second-generation metabolites (pMX-1 – pMX-n), each pMX corresponding to a first-generation metabolite and n ≤ 4. The probability threshold for these predictions was set at 20%. The main metabolic pathway predicted involved bromo-phenyl hydroxylation, followed by phase II conjugation reactions such as glucuronidation, sulfation, or methylation. Other predicted metabolites resulted from *N*-oxidation of the 1,4 diazepine, pyrrolidine ring oxidation, and hydroxylation of the pyrrolidine ring, and subsequent *O*-sulfation. *N*-Dealkylation at the diazepine ring, and a combination of *N*-dealkylation and carboxylation at the bromo-phenyl moiety were also forecasted. The structures, proposed biotransformations, and SMILES notations for in silico predicted metabolites of gidazepam and desalkylgidazepam are presented in Tables S3 and S4, respectively.

### HRMS/MS fragmentation of gidazepam and desalkylgidazepam

Gidazepam was identified in hepatocyte incubates and blood specimen with precursor ion at *m/z* 387.0454 ([M + H]^+^, C_17_H_16_BrN_4_O_2_^+^, 0.7 ppm), and retention time at 12.20 min. Fragmentation of the precursor ion produced a fragment ion (FI) at *m/z* 355.0078 (C_17_H_12_BrN_2_O_2_^+^, 0.4 ppm) resulting from a cleavage of the hydrazine group. This fragment may undergo further transformation through the sequential loss of two carbon monoxide molecules, forming FI at *m/z* 327.0127 (C_16_H_12_BrN_2_O^+^, − 0.2 ppm) and *m/z* 299.0178 (base peak, C_15_H_12_BrN_2_^+^, − 0.1 ppm), respectively. From *m/z* 299.0178, a further loss of hydrogen bromide could generate yet another fragment at *m/z* 219.0918 (C_15_H_11_N_2_^+^, 0.6 ppm). Alternatively, the FI at *m/z* 355.0078 could follow a different pathway involving *N*-desalkylation, possibly resulting in a minor fragment at *m/z* 315.0126 (C_15_H_12_BrN_2_O^+^, − 0.5 ppm) and downstream fragments at *m/z* 299.0178 and 219.0918.

Desalkylgidazepam was likewise detected in both hepatocyte incubates, and the blood sample analyzed by HRMS. The precursor ion was observed at *m/z* 315.0128 ([M + H]^+^, C_15_H_12_BrN_2_O^+^, 0.2 ppm). FI at *m/z* 287.0176 (C_14_H_12_BrN_2_^+^, − 0.8 ppm) might correspond to a loss of carbon monoxide. From this fragment, several subsequent fragmentation pathways are possible. Firstly, the loss of a bromine radical might lead to *m/z* 208.0995 (C_14_H_12_N_2_^+●^, 0.1 ppm). A further loss of a CH_3_N^●^ group may then produce FI at *m/z* 180.0808 (C_13_H_10_N^+^, 0.1 ppm). Secondly, an alternative pathway might involve the direct loss of the phenyl ring, resulting in a fragment at *m/z* 208.9709 (C_8_H_6_BrN_2_^+^, 0.1 ppm). A subsequent loss of a bromine radical from this species could lead to FI at *m/z* 130.0526 (C_8_H_6_N_2_^+^, 0.4 ppm).

### Gidazepam metabolites in human hepatocytes

**Fig. 1:**
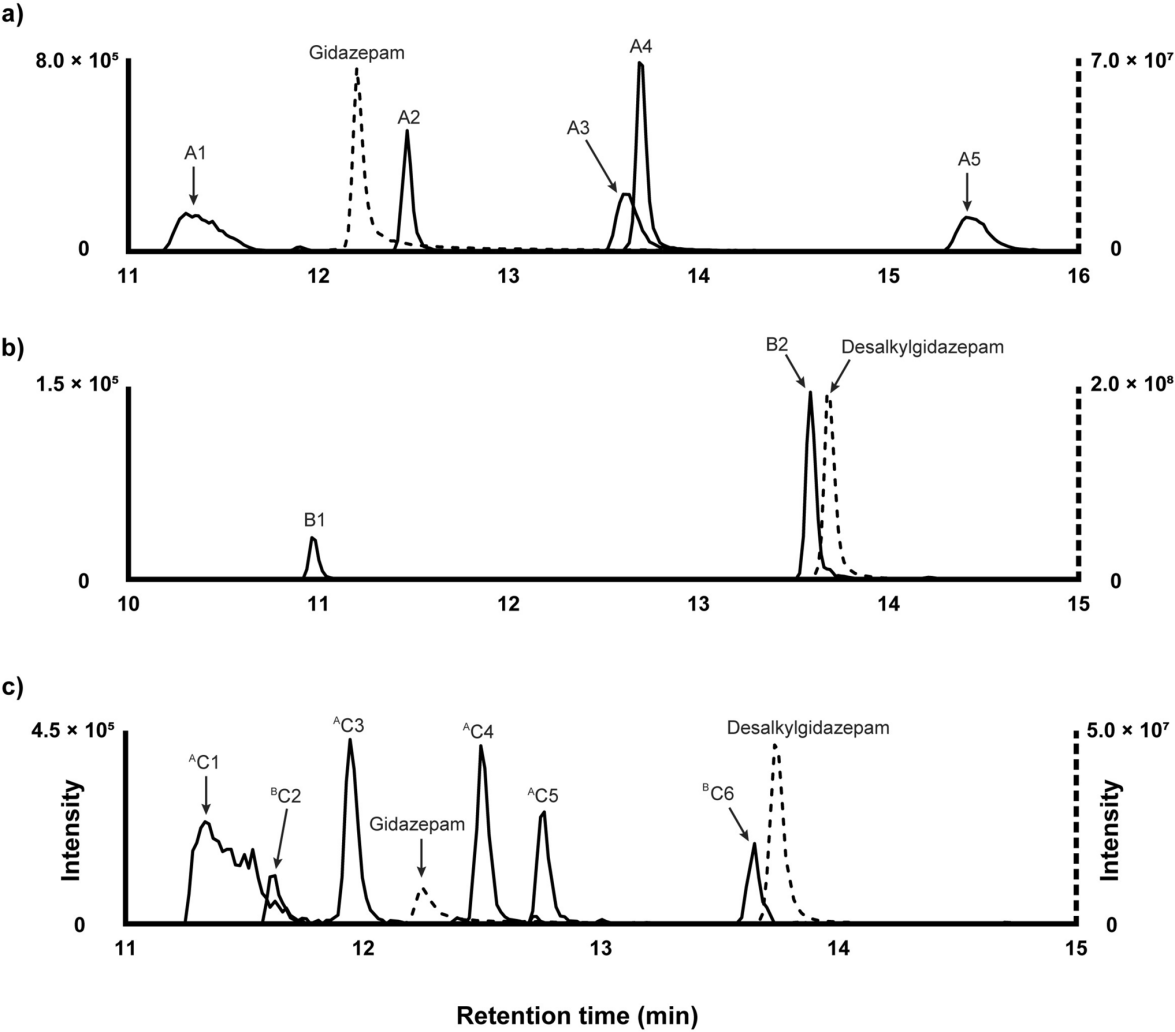
Extracted ion chromatographs of gidazepam (**a**) and desalkylgidazepam (**b**) and their metabolites in three hour human hepatocyte incubations and authentic human blood sample (**c**)

Figure 1a illustrates the chromatographic elution profiles of gidazepam and its metabolites identified after three hour incubation with human hepatocytes. The proposed biotransformations, chemical structures, chromatographic peak areas, and ∆ppm values for the identified metabolites are presented in Table 1.

**Table 1:** Proposed metabolic biotransformations, and elucidated elemental compositions, retention times, accurate molecular masses, mass errors, and chromatographic peak areas of gidazepam, desalkylgidazepam, and their metabolites after three hour human hepatocyte incubations and analysis using high-resolution mass spectrometry

ID	RT (min)	Metabolic transformation	Elemental composition	[M+H]^+^ (*m/z* ) [M-H]^-^ (*m/z*)	Mass error (∆ppm)	Peak area, 3 h incubation
*Gidazepam*						
A1	11.30	*N*-Glucuronidation	C_23_H_23_BrN_4_O_8_	563.0769 561.0648	− 0.54 3.83	2.5 × 10^6^ 1.7 × 10^6^
Gidazepam	12.20	Parent	C_17_H_15_BrN_4_O_2_	387.0454 385.0320	0.74 3.74	3.7 × 10^8^ 5.1 × 10^6^
A2	12.47	*N*-Acetylation	C_19_H_17_BrN_4_O_3_	429.0555 427.0427	− 0.42 3.69	1.9 × 10^6^ 6.8 × 10^5^
A3	13.60	Two fold *N*-Acetylation+ Carboxylation	C_22_H_19_BrN_4_O_6_	515.0566 513.0432	1.02 − 3.49	2.1 × 10^6^ 1.7 × 10^6^
A4	13.69	*N*-Desalkylation	C_15_H_11_BrN_2_O	315.0127 –	− 0.17 –	3.5 × 10^6^ –
A5	15.41	Two-fold *N*-Acetylation	C_21_H_19_BrN_4_O_4_	471.0664 469.0531	0.54 3.00	1.7 × 10^6^ 1.6 × 10^6^
*Desalkylgidazepam*						
B1	10.96	Hydroxylation + *O*-Glucuronidation	C_21_H_19_BrN_2_O_8_	507.0402 505.0269	0.89 3.36	1.2 × 10^6^ 7.0 × 10^5^
B2	13.66	Hydroxylation	C_15_H_11_BrN_2_O_2_	331.0077 328.9943	0.10 3.61	5.8 × 10^6^ 1.5 × 10^6^
Desalkylgidazepam	13.73	Parent	C_15_H_11_BrN_2_O	315.0128 312.9995	0.15 4.16	8.9 × 10^8^ 8.1 × 10^5^

The earliest eluting metabolite, designated A1, was observed at 11.30 min with a precursor ion at *m/z* 563.0769, ([M + H]^+^, C_23_H_24_BrN_2_O_8_^+^, − 0.5 ppm), suggesting* N*-glucuronidation. The MS/MS spectrum had FI at *m/z* 387.0442 (C_17_H_16_BrN_4_O_2_^+^, − 2.4 ppm), which may correspond to the gidazepam core following the loss of the conjugate group (− 176.0327 u). Additional fragments of A1 resembled those of the parent compound. For instance, the potential loss of a hydrazine group could yield *m/z* 355.0059 (C_17_H_12_BrN_2_O_2_^+^, -5.0 ppm), while two consecutive carbon monoxide losses could produce fragments *m/z* 327.0128 (C_16_H_12_BrN_2_O^+^, 0.1 ppm) and *m/z* 299.0170 (C_15_H_12_BrN_2_^+^, − 2.8 ppm).

A2 eluted immediately after gidazepam at 12.47 min. The precursor ion at *m/z* 429.0555 ([M + H]^+^, C_19_H_18_BrN_4_O_3_^+^, − 0.4 ppm) indicates *N*-acetylation reaction. Acetyl group cleavage likely yields FI at *m/z* 387.0441 (C_17_H_16_BrN_4_O_2_^+^, − 2.6 ppm), consistent with protonated gidazepam. Moreover, the MS/MS spectrum of A2 closely mimicked that of the parent compound, with both displaying a base peak at *m/z* 299.0171 (C_15_H_12_BrN_2_^+^, -2.5 ppm), indicating analogous fragmentation pattern.

The next eluting metabolite, A3 likely had two acetyl groups and one carboxyl modifications, based on the precursor ion at *m/z* 515.0566 ([M + H]^+^, C_22_H_20_BrN_4_O_6_^+^, 1.0 ppm), and was detected at 13.60 min. A subsequent loss of carbon dioxide likely generates a fragment at *m/z* 471.0642 ([M + H]^+^, C_21_H_20_BrN_4_O_4_^+^, − 4.3 ppm). Stepwise fragmentation of the conjugated acetylated hydrazine chain could produce FI at *m/z* 355.0082 (C_17_H_12_BrN_2_O_2_^+^, 1.5 ppm), after which the fragmentation pattern appears to follow that of gidazepam.

A4 was also detected at 13.69 min, with a product ion at *m/z* 315.0127 ([M + H]^+^, C_15_H_12_BrN_2_O^+^, − 0.2 ppm), showed a fragmentation pattern closely resembling that of desalkylgidazepam, as described previously.

Finally, A5 eluted at 15.41 min and the observed product ion at *m/z* 471.0664 ([M + H]^+^, C_21_H_20_BrN_4_O_4_^+^, 0.5 ppm) is consistent with a two-fold acetylation. A combined carbon dioxide could produce FI at *m/*z 427.0773 (C_20_H_20_BrN_4_O_2_^+^, 2.1 ppm), followed by the loss of the remaining hydrazine chain, resulting in *m/z* 355.0064 (C_17_H_12_BrN_2_O_2_^+^, − 3.6 ppm). From this fragment onward, the fragmentation pattern of A5 aligns with those identified for A2 and gidazepam. Key fragments include *m/z* 327.0119 (C_16_H_12_BrN_2_O^+^, − 2.6 ppm), *m/z* 299.0171 (also base peak, C_15_H_12_BrN_2_^+^, − 2.5 ppm), and *m/z* 219.0912 (C_15_H_11_N_2_^+^, − 2.1 ppm).

### Desalkylgidazepam metabolites in human hepatocytes

Table 1 details the proposed metabolic transformations, tentative chemical structures, chromatographic peak areas, and ∆ppm values for desalkylgidazepam and its metabolites. Figure 1b depicts the chromatogram of desalkylgidazepam and its metabolites after three hours of incubation with human hepatocytes.

B1, detected at 10.96 min, with precursor mass at *m/z* 507.0402 ([M + H]^+^, C_21_H_20_BrN_2_O_8_^+^, 0.9 ppm), had a mass shift of + 192.0274 u compared to desalkylgidazepam. This shift could be explained by *O*-glucuronidation following hydroxylation. The fragmentation spectrum indicates loss of the conjugate, resulting in a base peak at *m/z* 331.0064 (C_15_H_12_BrN_2_O_2_^+^, − 3.8 ppm). Tentative interpretation of the mass data suggests a further loss of carbon monoxide yields FI at *m/z* 303.0127, although this fragment was not observed. Starting from this hypothetical fragment, three potential pathways might occur: firstly, a loss of a bromine radical could yield *m/z* 224.0929 (C_14_H_12_N_2_O^+^, − 6.8 ppm); secondly, FI at *m/z* 224.9645 (C_8_H_6_BrN_2_O^+●^, − 5.8 ppm) may be attributed to a loss of the phenyl ring; and thirdly, a loss of benzonitrile could potentially result in a fragment at *m/z* 199.9701 (C_7_H_7_BrNO^+^, − 2.3 ppm). The absence of a water loss may suggest aromatic hydroxylation rather than hydroxylation at the 3-position of the diazepine ring.

B2 was detected at 13.66 min. The precursor ion at *m/z* 331.0077 ([M + H]^+^, C_15_H_12_BrN_2_O_2_^+^, 0.1 ppm) featured a mass shift of + 15.995 u relative to desalkylgidazepam, which corresponds to hydroxylation. The MS/MS spectrum indicates a potential water loss, resulting in FI at *m/z* 312.9966 (C_15_H_10_BrN_2_O^+^, − 1.6 ppm). A subsequent carbon monoxide loss appeared to yield the base peak at *m/z* 285.0017 (C_14_H_10_BrN_2_^+^, − 1.7 ppm), followed by a further bromine radical loss, producing *m/z* 205.0760 (C_14_H_9_N_2_^+●^, − 0.1 ppm). Alternatively, the fragment at *m/z* 275.0177 (C_13_H_12_BrN_2_^+^, − 0.5 ppm) might result from a direct loss of two carbon monoxide molecules, with a further hydrogen bromide loss leading to *m/z* 195.0918 (C_13_H_11_N_2_^+^, 0.6 ppm). A minor fragment at *m/z* 224.9653 (C_8_H_6_BrN_2_O^+^, − 2.2 ppm) may indicate a loss of the phenyl ring, whereas an additional water loss may account for FI at *m/z* 206.9548 (C_8_H_4_BrN_2_^+^, − 2.1 ppm). Taken together, the varying fragmentation pathways of metabolites B1 and B2 may reflect hydroxylation occurring at different sites, with B2 likely hydroxylated at position 3 of the diazepine ring.

### Metabolites detected in blood

Altogether, six metabolites of gidazepam and desalkylgidazepam were identified in blood; their details are summarized in Table 2. Figure 1c shows the elution profiles of gidazepam, desalkylgidazepam, and their metabolites in blood following UHPLC-HRMS analysis. Three of these metabolites, namely C1 (A1), C4 (A2), and desalkylgidazepam (A4), found in blood were also detected after three hour in vitro incubation with human hepatocytes and gidazepam. Additionally, C6 (corresponding to B2) was observed in both blood and desalkylgidazepam incubations with hepatocytes.

**Table 2:** Gidazepam, desalkylgidazepam, and their metabolites: predicted chemical structures, retention times, accurate molecular masses, mass errors, and peak areas identified in a human biosample analyzed with UHPLC-HRMS/MS

ID	RT (min)	Metabolic transformation	Elemental composition	[M+H]^+^ (m/z) [M-H]^−^ (m/z)	Mass error(∆ppm)	Peak area, blood
^A^C1	11.40	*N*-Glucuronidation	C_23_H_23_BrN_4_O_8_	563.0777 561.0648	0.88 3.83	3.2 × 10^6^ 1.6 × 10^6^
^B^C2	11.63	Hydroxylation + *O*- Glucuronidation	C_21_H_19_BrN_2_O_8_	507.0398 505.0260	0.09 1.58	1.8 × 10^5^ 3.5 × 10^5^
^A^C3	11.94	*N*-Glucuronidation	C_23_H_23_BrN_4_O_8_	563.0779 561.0645	1.24 3.30	1.8 × 10^6^ 6.4 × 10^6^
Gidazepam	12.25	Parent	C_17_H_15_BrN_4_O_2_	387.0454 385.0318	0.74 3.22	6.8 × 10^7^ 2.5 × 10^5^
^A^C4	12.49	*N*-Acetylation	C_19_H_17_BrN_4_O_3_	429.0558 427.0422	0.28 2.51	1.6 × 10^6^ 3.5 × 10^5^
^A^C5	12.76	Unknown	C_19_H_18_BrN_3_O_3_	416.0606 414.0461	0.41 0.54	1.0 × 10^6^ 1.5 × 10^4^
^B^C6	13.65	Hydroxylation	C_15_H_11_BrN_2_O_2_	331.0077 328.9939	0.10 2.39	6.8 × 10^5^ 9.6 × 10^4^
Desalkylgidazepam	13.78	Parent	C_15_H_11_BrN_2_O	315.0129 –	0.47 –	2.2 × 10^8^ –

C2 shared the same product ion as B1 (*m/z* 507.0402), suggesting a similar biotransformation to that proposed for B1. C2’s MS/MS spectrum was similar to that of B2, showing intense FIs at *m/z* 312.9978, *m/z* 285.0017 (base peak), *m/z* 238.0734, *m/z* 205.0763, and *m/z* 104.0495, pointing towards O-glucuronidation following hydroxylation in position 3 of the diazepine ring.

The MS/MS spectrum of C3, identified at 11.94 min and *m/z* 563.0779, closely matched that of C1 (A1), corresponding to *N*-glucuronidation reaction. C3 also showed a base peak at *m/z* 299.0175, with additional fragments at *m/z* 355.064, *m/z* 327.0133, *m/z* 219.0915, and *m/z* 203.0735 all consistent with the fragmentation pattern of C1 and gidazepam.

Lastly, C5, identified at 12.76 min and with product ion at *m/z* 416.0606, loses the hydrazine group at FI *m/z* 355.0067 (C_17_H_12_BrN_2_O_2_^+^, − 2.7 ppm), and subsequent loss of carbon monoxide producing *m/z* 327.0114 (C_16_H_12_BrN_2_O^+^, − 4.1 ppm). The most intense fragment was observed at *m/z* 299.0170 (C_15_H_12_BrN_2_^+^, − 2.8 ppm), with other fragments detected at *m/z* 281.9913 (C_15_H_9_BrN^+^, 0.1 ppm), *m/z* 219.0911 (C_15_H_11_N_2_^+^, − 2.6 ppm), and *m/z* 203.0728 (C_15_H_9_N^+^, − 0.7 ppm).

### GABAAR and TSPO 18 kDa pharmacodynamic modelling* in silico*

**Fig. 2:**
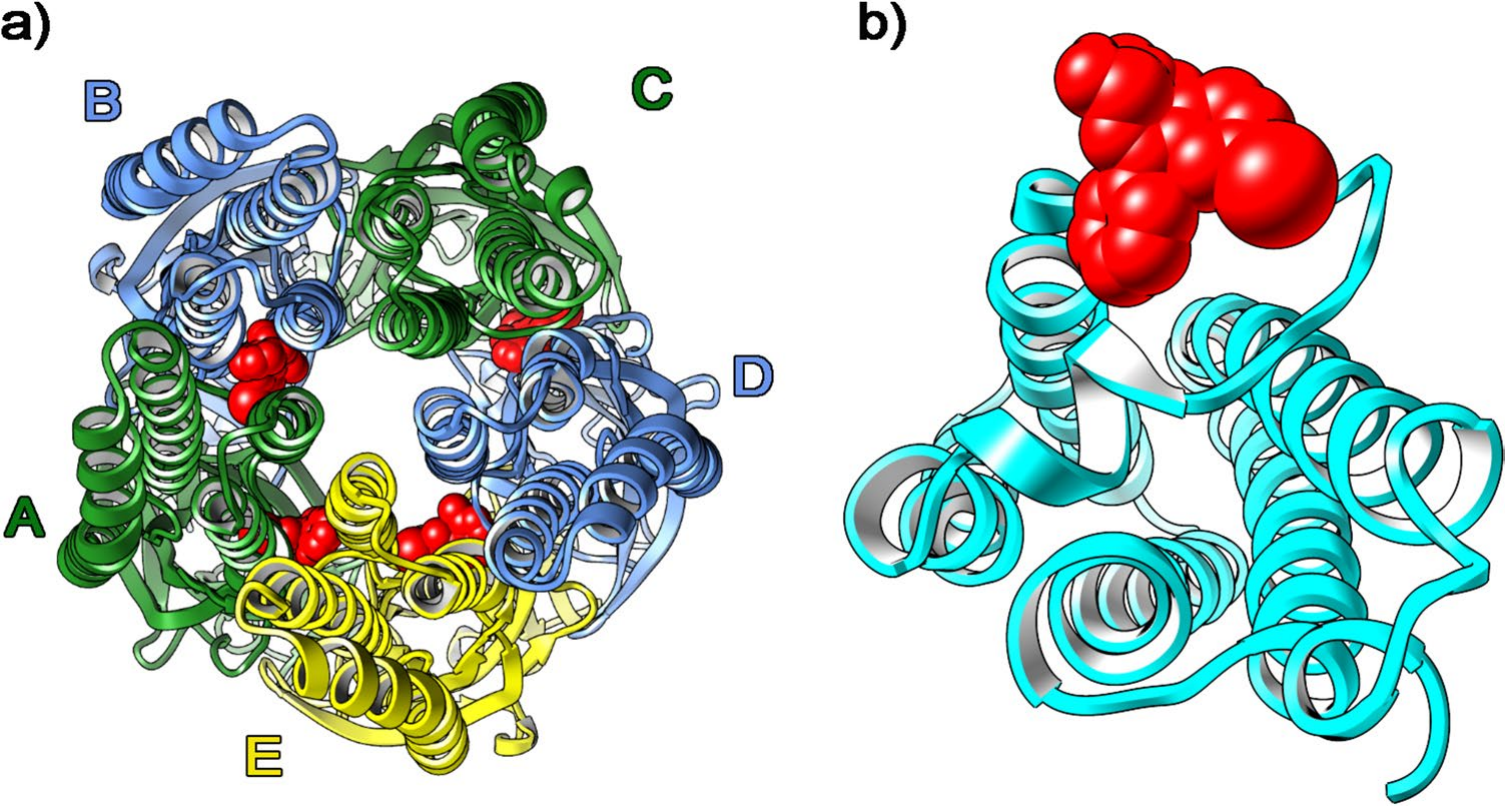
Overview of binding sites for benzodiazepines investigated on γ-amino butyric acid A receptor (GABA_A_R) (**a**) following focused docking approach, and the univocal binding site of 18 kDa translocator protein (**b**) reported in cyan ribbons. The α, β, and γ subunits of GABA_A_R are reported in green, blue, and yellow ribbons, respectively

The main site for benzodiazepine activity on GABA_A_R is the α_1_γ_2_ interface, albeit other sites were found for gidazepam and desalkylgidazepam. TSPO, however, had a univocal binding spot for these compounds. Figure 2 depicts the binding sites identified for all benzodiazepines investigated including diazepam and 4-chlorodiazepam on GABA_A_R (a) and TSPO (b). Results from the in silico modelling of gidazepam, desalkylgidazepam, (3*R*)- and (3*S*)-hydroxy desalkylgidazepam, diazepam, and 4-chlorodiazepam are summarized in Table 3.

**Table 3:** Predicted binding energies (kcal/mol), inhibition constants and K_i_ (nM) values calculated following “targeted” molecular docking on GABA_A_R and TSPO 18 kDa for gidazepam, desalkylgidazepam, and (3*R*)-/(3*S*)-hydroxy desalkylgidazepam

Ligand/receptor	GABA-A			TSPO	
	Binding energy	K_i_	Subunit	Binding energy	K_i_
Gidazepam	− 9.89	2400	α_1_/γ_2_ (A/E)	− 12.54	0.647
	− 10.09	1800	α_1_/β_2_ (A/B)		
	− 9.98	2300	α_1_/β_2_ (C/D)		
Desalkylgidazepam	− 10.81	12.1	α_1_/γ_2_ (A/E)	− 12.84	0.389
	− 7.93	2540	γ_2_/β_2_ (E/D)		
(3*R*)-Hydroxy desalkylgidazepam	− 10.28	15.2	α_1_/γ_2_ (A/E)	− 12.52	0.661
(3*S*)-Hydroxy desalkylgidazepam	− 10.79	13.9	α_1_/γ_2_ (A/E)	− 12.51	0.673
Diazepam	− 10.53	14.6	α_1_/γ_2_ (A/E)	− 12.12	1.28
	− 8.29	2850	α_1_/ β_2_ (A/B)		
	− 7.72	3930	γ_2_/β_2_ (E/D)		
4-Chlorodiazepam	− 5.29	107,140	α_1_/γ_2_ (A/E)	− 12.24	1.06

### Molecular docking and dynamics simulations on GABAAR

**Fig. 3:**
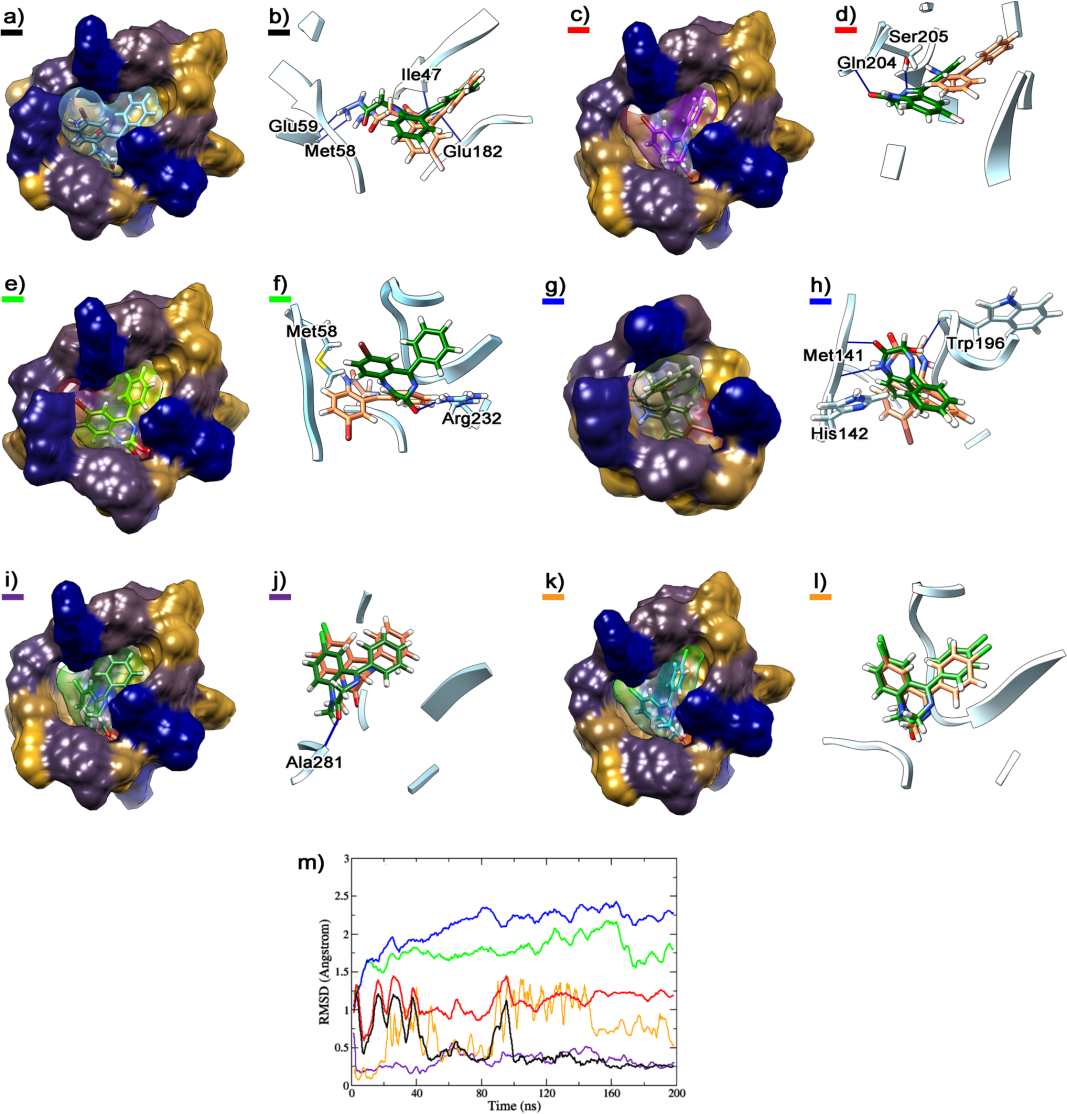
Binding mode of gidazepam (**a**, **b**), desalkylgidazepam (**c**, **d**), (3*R*)- hydroxy desalkylgidazepam (**e**, **f**), (3*S*)-hydroxy desalkylgidazepam (**g**, **h**), diazepam (**i**, **j**), and 4-chlorodiazepam (**k**, **l**) on α_1_γ_2_ subunit of γ-amino butyric acid A receptor (GABA_A_R). The starting pose and the final pose after MD are reported in coral and dark green sticks, respectively, the residues of GABA_A_R interacting are underlined in light blue ribbons, and the H-bonds are depicted with blue lines. The volume of the α_1_γ_2_ binding site is highlighted in surface with a color scale ranging from navy blue for highly hydrophilic amino acid regions, to goldenrod for extremely non-polar amino acids. The surfaces of the compounds are reported with transparency. The RMSD profiles (i) of gidazepam (black), desalkylgidazepam (red), (3*R*)- hydroxy desalkylgidazepam (green), (3*S*)-hydroxy desalkylgidazepam (blue), diazepam (purple), and 4-chlorodiazepam (orange), in the binding sites at α_1_γ_2_ interface, are also depicted

Gidazepam formed a hydrogen bond, via its carbonyl group, with the amine group of Ala281 backbone at the α_1_γ_2_ site and its hydrazine moiety was oriented towards a non-polar amino acid region. Similarly, desalkylgidazepam and its 3-hydroxy metabolites also established a hydrogen bond between their carbonyl group and the amine group of Ala281. Additionally, the (3*R*)-hydroxy desalkylgidazepam metabolite, through its hydroxyl group, interacted with the side chain of Arg232 via hydrogen bonding. In contrast, the (3*S*)-hydroxy counterpart formed one and two hydrogen bonds with Trp196 and Met141, respectively. Figure 3 depicts the binding modes of gidazepam, desalkylgidazepam, (3*R*)- and (3*S*)-hydroxy desalkylgidazepam, diazepam, and 4-chlorodiazepam at the α_1_γ_2_ interface. Diazepam, notably, shows similar RMSD values as gidazepam, albeit reaching stabilization faster than this compound. On the other hand, 4-chlorodiazepam exhibited very noticeable oscillations and did not converge to predefined values, indicating a significantly lower binding affinity than other compounds investigated.

**Fig. 4:**
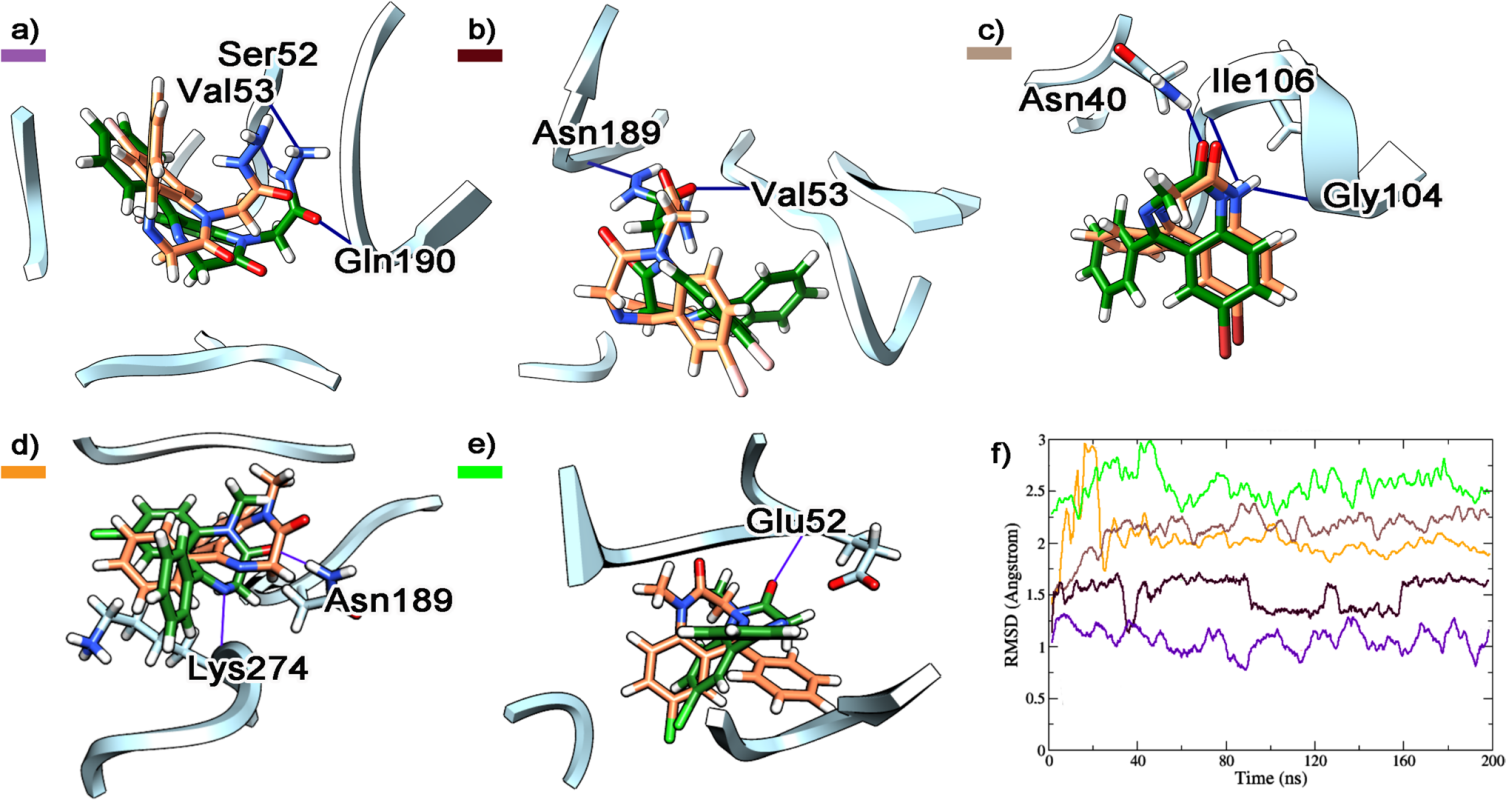
Depiction of gidazepam binding at α_1_β_2_ subunits illustrating interactions at the A/B interface (**a**) and C/D interface (**b**), desalkylgidazepam’s interaction at γ_2_β_2_, E/D interface (**c**) and diazepam’s binding mode at α_1_β_2_ (**d**) and γ_2_β_2_ (**e**) subunits corresponding to A/B and E/D chains, respectively. The starting and final poses after MD are reported in coral and dark green sticks, respectively, and the interacting residues of GABA_A_R are underlined in light blue ribbons and H-bonds are depicted with blue lines. RMSD plots (**f**) of gidazepam (purple and deep brown), desalkylgidazepam (light brown), and diazepam (orange and green) are reported

Additional binding sites for gidazepam were identified at two α_1_β_2_ interfaces. In the pose at the α_1_β_2_ site (A/B chains), gidazepam was located internally within the calyx formed by the pentameric structure of the GABA_A_R, in proximity to the transmembrane domains (TMD). Here, gidazepam interacted via hydrogen bonds with Ser52, Val53, and Gln190. At the α_1_β_2_ site involving the C/D subunits, hydrogen bonds with Val53 and Asn189 were observed. The RMSD profiles for gidazepam at these two binding sites were 1.046 ± 0.027 Å (A/B chains) and 1.643 ± 0.022 Å (C/D chains) after 150 ns. Furthermore, an additional binding pose for desalkylgidazepam was identified at the β_2_γ_2_ interface, where it formed hydrogen bonds with Gln204 and the side chain of Ser205. Desalkylgidazepam, however, exhibited slow stabilization within its binding site, with an average RMSD of 2.312 ± 0.018 Å, reaching a steady state after 160 ns. For diazepam, molecular modelling revealed a pose at α_1_β_1_ site, having two H-bonds with Asn189 and Lys274, and another pose at β_2_γ_2_ interface, stabilized via H-bonding with Glu52. No additional binding sites were observed for either (3*R*)- or (3*S*)-hydroxy desalkylgidazepam. Figure 4 presents the other binding modes identified for gidazepam, desalkylgidazepam, and diazepam through molecular modelling.

### Molecular docking and dynamics simulations on TSPO 18 kDa receptor

Gidazepam, (3*R*)-, and (3*S*)-hydroxy desalkylgidazepam anchored to the TSPO binding site with the POPC system and maintained the binding pose throughout MD simulations. Desalkylgidazepam, however, was completely solvated after 60 ns. Gidazepam showed two H-bonds with the receptor complex, three H-bonds with water molecules, and a salt bridge between the hydrazine and the POPC’s phosphate group. For the (3*R*)-hydroxy metabolite, Arg43 directly interacted through π-alkyl interaction with the compound and an H-bond with oxygen from the phosphate of the POPC residue were observed. Six H-bonds with water molecules were observed, compared to the three found for gidazepam. The (3*S*)-hydroxy enantiomer, on the other hand, demonstrated a lower affinity for the TSPO-POPC membrane after the MD simulation, with fewer interacting amino acids. In this case, Lys36 through π-alkyl interaction, in place of Arg43, oriented towards the protein was found. Gidazepam, (3*R*)-, and (3*S*)-hydroxy desalkylgidazepam’s RMSD values on the TSPO with POPC membrane were 0.64 ± 0.031 Å, 0.66 + 0.008 Å, and 0.64 ± 0.008 Å, respectively.

**Fig. 5:**
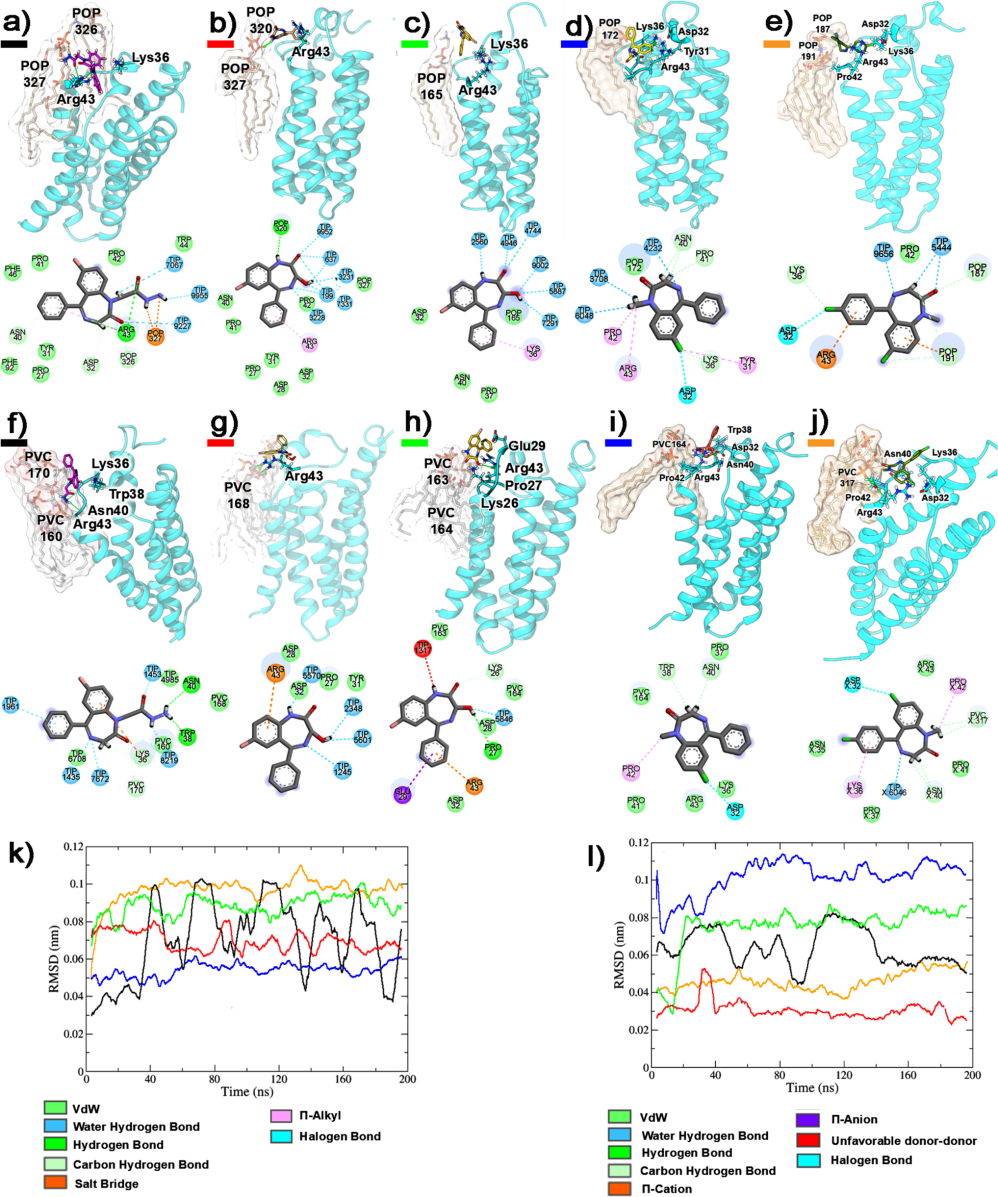
Illustration of the binding mode of gidazepam (**a**), (3*R*)-hydroxy desalkylgidazepam (**b**), (3*S*)-hydroxy desalkylgidazepam (**c**), diazepam (**d**), and 4-chlorodiazepam (**e**) on the 18 kDa translocator protein (TSPO) in the presence of 1-Palmitoyl-2-oleoyl-glycero-3-phosphocholine (POPC). The binding mode with TSPO in presence of 1,1’-Palmitoyl-2,2’-vacenoyl-cardiolipin (PVCL2) for gidazepam (**f**), (3*R*)-hydroxy desalkylgidazepam (**g**), (3*S*)-hydroxy desalkylgidazepam (**h**), diazepam (**i**), and 4-chlorodiazepam (**j**) are also depicted. RMSD values in presence of POPC (**k**) and PVCL2 (**l**) lipids are also shown for gidazepam (black), (3*R*)-hydroxy desalkylgidazepam (red), (3*S*)-hydroxy desalkylgidazepam (green), diazepam (blue), and 4-chlorodiazepam (orange)

Desalkylgidazepam again was unable to maintain the binding site with the TSPO-PVCL2 membrane and was completely solvated after 70 ns. Gidazepam generated five H-bonds with water molecules and two H-bonds with Trp38 and Asn40. Additional H-bond with Arg43 of the proximal PVCL2 residue, was also observed. The (3*R*) enantiomer directly interacted with Arg43 through π-cation interaction, and with Asp32 through an H-bond. Bonding with four water molecules were also involved, and a Van der Waals interaction with a PVCL2 residue was found. For the (3*S*) enantiomer, π-cation interaction with Arg43, two H-bonds with Asp28 and Pro27, and Van der Waals interactions with two PVCL2 residues were prominent. Only one H-bond with water was observed, and π-anion interaction with Glu29 was also detected. The RMSD of the compounds in presence of PVCL2 were gidazepam, 0.62 ± 0.019 Å; (3*R*)-hydroxy desalkylgidazepam, 0.28 ± 0.004 Å; (3*S*)-hydroxy desalkylgidazepam, 0.77 ± 0.009 Å. Figure 5 depicts the binding modes of gidazepam, (3*R*)-, (3*S*)-hydroxy desalkylgidazepam, diazepam, and 4-chlorodiazepam on the TSPO with POPC and PVCL2 membranes.

## Discussion

### Comparing* in vitro* and* in vivo* gidazepam and desalkylgidazepam metabolites

*N*-Desalkylation appears to be the primary biotransformational pathway of gidazepam, as reported in previous animal models and human studies (Merette et al., 2023). In three hour hepatocyte incubation with gidazepam, A4 was identified as desalkylgidazepam based on its retention time, molecular mass, and MS/MS fragmentation, consistent with desalkylgidazepam reference standard. Nonetheless, both in vitro and in vivo metabolism of gidazepam seems to involve further modifications to the hydrazine moiety. Specifically, these modifications led to the formation of *N*-glucuronides, as suggested by a + 176.03 u (+ C_6_H_8_O_6_) mass shift from gidazepam in both C1 (A1) and C3. The formation of C1 and C3 is likely facilitated by UDP-glucuronyltransferases, enzymes typically involved in glucuronide conjugation of xenobiotics (Court 2014). Additionally, C3 was also detected in hepatocyte incubations, albeit with a lower intensity. Further biotransformations include *N*-acetylation, as indicated for C4, while A5 could have undergone a two-fold *N*-acetylation at the hydrazine group. Interestingly, A3 seems to arise from an additional carboxylation of A5, as indicated by a mass difference of 43.9902 u (+ CO_2_). This biotransformation may render A3 more susceptible to renal excretion, although this metabolite has not yet been described in urine (Talevi and Bellera 2018; Gameli et al., 2024). A3 and A5 were only observed in hepatocyte incubations, which could be explained by individual variability, considering that only a single blood sample was available in this study. However, it is more likely that these metabolites are not produced in vivo, as the corresponding intermediate (*N*-acetyl gidazepam) is probably sufficiently polar to be rapidly excreted before further transformation. Accordingly, the formation of A3 and A5 in vitro may result from the elevated substrate concentrations in the incubation system.

Regarding C5, this metabolite was identified in blood but not found hepatocyte incubations, suggesting hepatic enzymes may not be involved in this biotransformation. The proposed molecular structure, C_19_H_18_BrN_3_O_3_, suggests a few transformational paths are plausible. First, it is likely to have deamination of C4 (A2) and subsequent reduction of the carbonyl to a hydroxyl. Alternatively, direct conjugation of an ethoxy moiety following deamination may have yield C5. Overall, C5 seems to undergo a biotransformational pathway that needs further experimentation to fully elucidate this reaction.

**Fig. 6:**
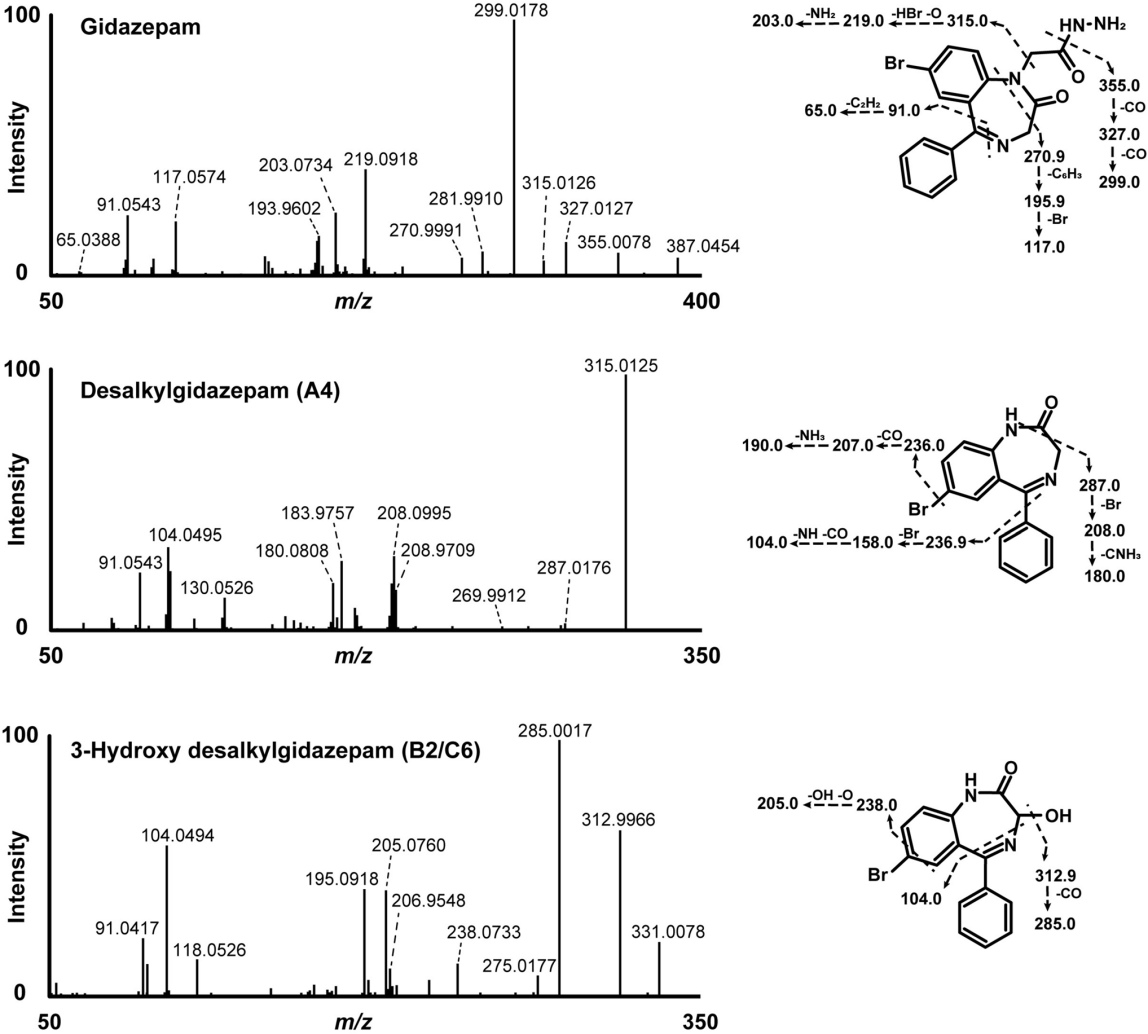
HRMS/MS spectra of gidazepam, desalkylgidazepam and 3-hydroxy desalkylgidazepam detected in three hour human hepatocyte incubations and blood sample analyzed with high-resolution mass spectrometry. Fragment ions in [] were anticipated or not detected in the MS/MS

**Fig. 7:**
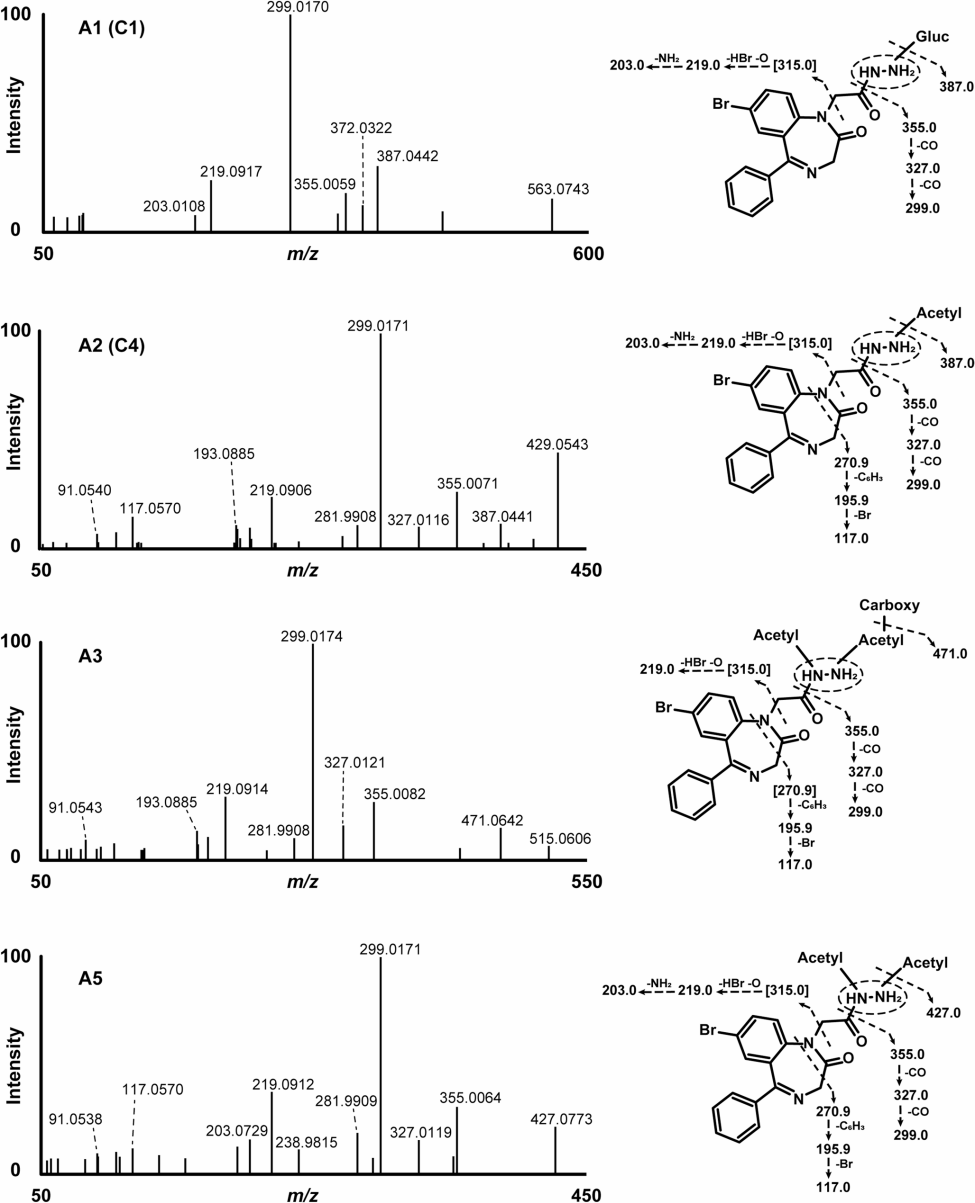
Fragmentation pattern of gidazepam metabolites detected in three hour human hepatocyte incubation. Fragment ions in [] were anticipated or not detected in the MS/MS. Carboxy, carboxylic acid; gluc, glucuronide

**Fig. 8:**
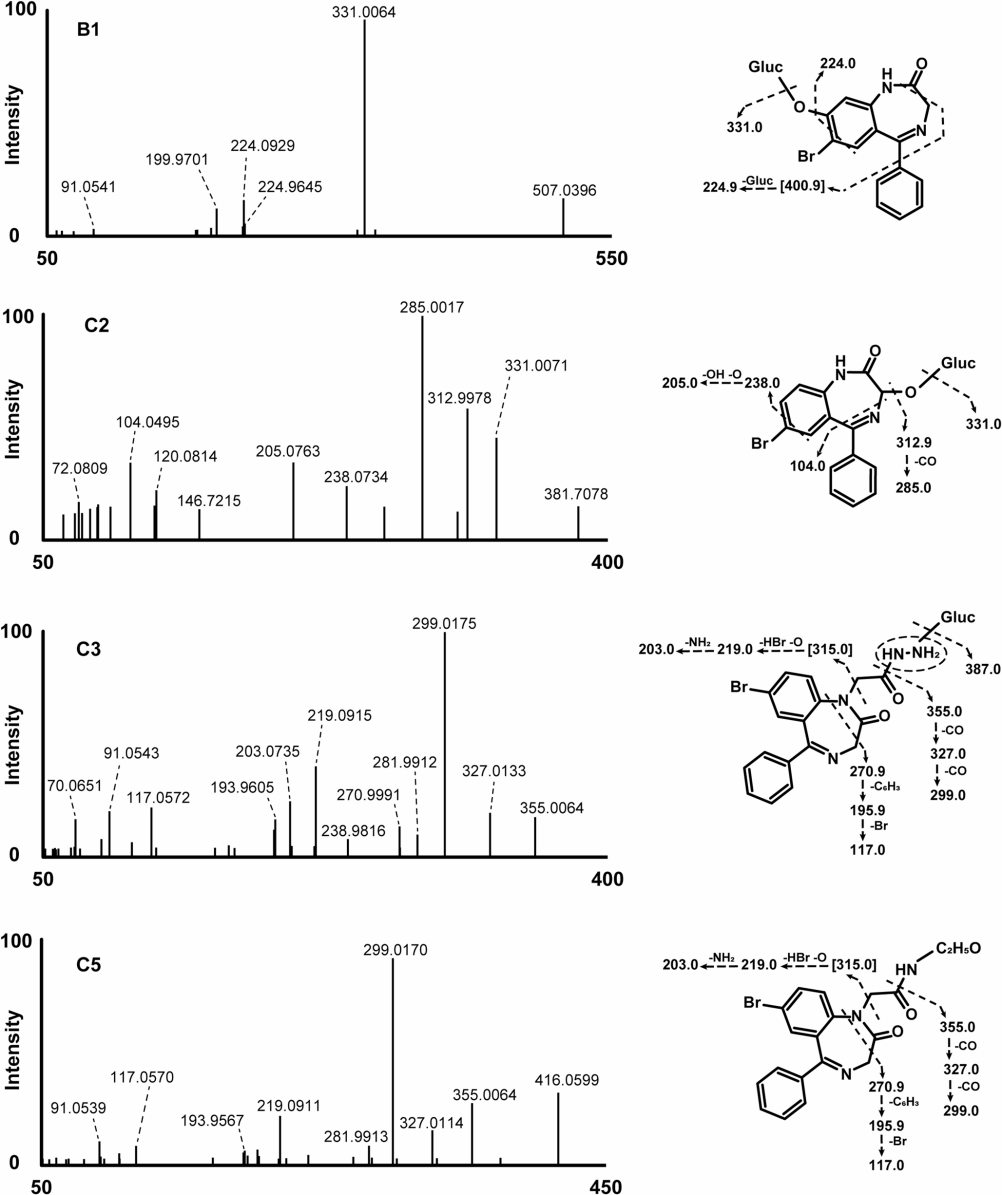
MS/MS of desalkylgidazepam metabolite in hepatocyte incubation (B1) and, other gidazepam and desalkylgidazepam metabolites identified in authentic blood sample following high-resolution mass spectrometry analysis. Fragment ion in [] were anticipated or not detected in the MS/MS. Gluc, glucuronide

**Fig. 9:**
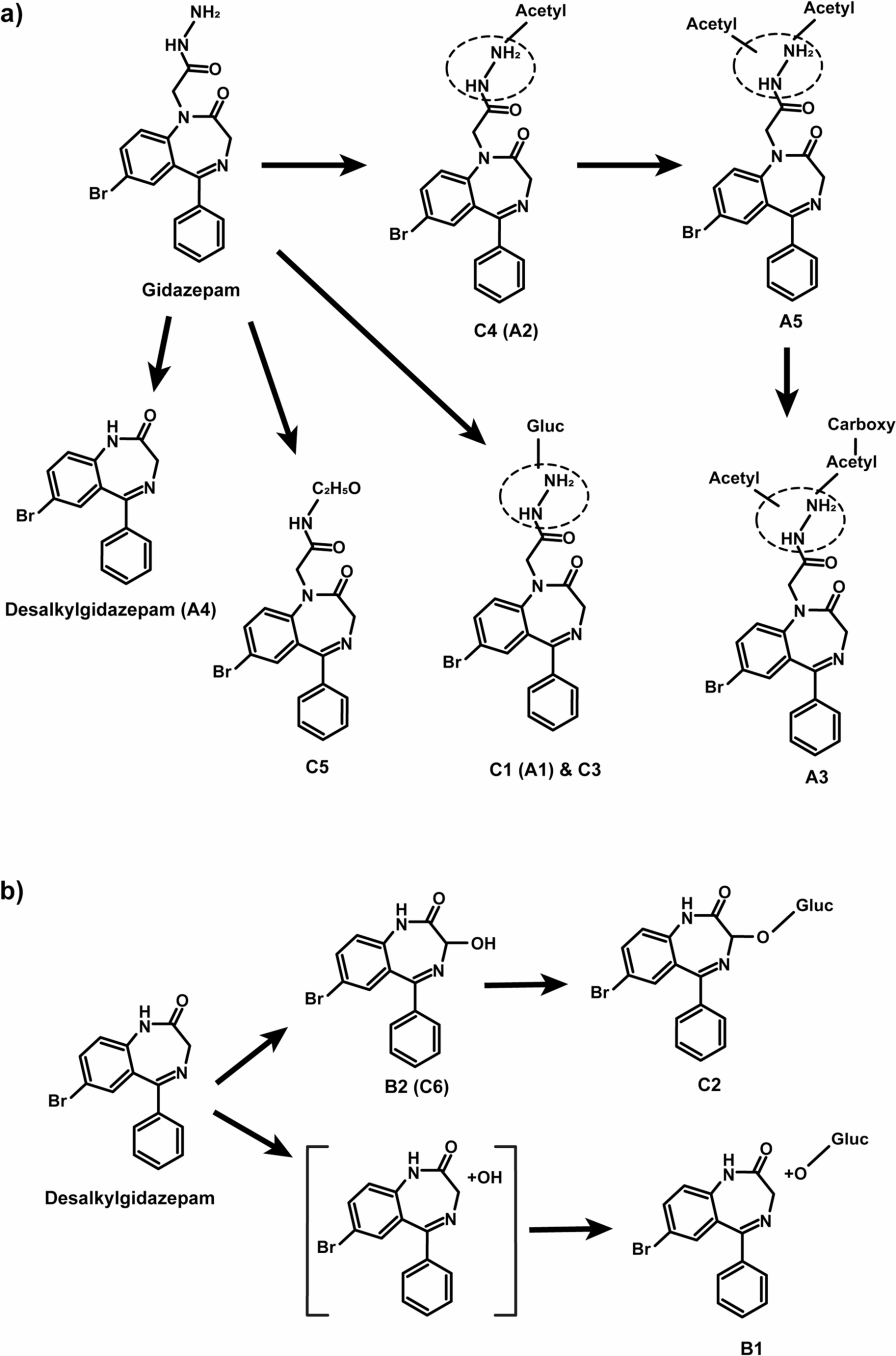
Proposed metabolic fate for gidazepam (**a**) and desalkylgidazepam (**b**) in human. Metabolite depicted in [] were anticipated or detected with low chromatographic peak areas. Gluc, glucuronide

Desalkylgidazepam appears to be primarily metabolized via hydroxylation, as suggested by + 15.995 u (+ O) shift. This biotransformation correlates with the active 3-hydroxy metabolite of desalkylgidazepam (B2). Figure 6 depicts the MS/MS spectra of gidazepam, desalkylgidazepam and 3-hydroxy desalkylgidazepam. The MS/MS fragmentation of all other metabolites identified following three hour human hepatocytes with gidazepam and desalkylgidazepam, and authentic blood sample are depicted in Figs. 7 and 8. C2 detected at 11.63 min, tentatively assigned as the phase II glucuronide of B2, was found in higher intensity in blood than hepatocyte incubations. Two other hydroxy desalkylgidazepam metabolites, with minor peak areas, were observed at 9.93 and 12.35 min, and plausibly, their respective phase II glucuronides were at 8.22 and 10.96 min (B1) in hepatocyte incubation. Figure 9 shows the proposed biotransformation pathways of gidazepam and desalkylgidazepam.

### Gidazepam and desalkylgidazepam consumption markers

Gidazepam-*N*-glucuronide and *N*-acetyl gidazepam appeared to be the predominant metabolites in hepatocyte incubation and blood. Since desalkylgidazepam is available on the NPS market, it is necessary to detect both gidazepam-*N*-glucuronide and *N*-acetyl gidazepam along with the parent compound in blood to strengthen the evidence for gidazepam consumption. In cases involving suspected desalkylgidazepam intoxication, retrospective data analysis for gidazepam-specific markers might help to exclude gidazepam use, as desalkylgidazepam and its abundant metabolites could be produced downstream following gidazepam administration.

Additionally, carboxymethylgidazepam reportedly the most abundant urine metabolite, approximately five times more prevalent than desalkylgidazepam (Maskell et al., 2023), was found in both 0 h and 3 h hepatocyte incubations at similar intensities. Interestingly, in buffer controls incubated for three hours and blood sample analyzed, carboxymethylgidazepam’s peak area was ten times the area found for 3 h hepatocytes. The present data thus suggests carboxymethylgidazepam formation is not mediated by hepatocytes and may be a degradation byproduct or metabolite formed in other context. Nonetheless, inclusion of this compound could prove valuable for both clinical and forensic analysis. In the present context, the relatively high peak area for gidazepam and desalkylgidazepam might indicate co-consumption of both substances, although desalkylgidazepam’s relatively high volume of distribution suggests it may undergo postmortem redistribution (Maskell et al., 2023). This may explain the high amount of desalkylgidazepam identified in LC–MS/MS and LC-HRMS analysis.

### *In silico* pharmacodynamics at the GABAAR

Gidazepam demonstrated a low binding affinity at GABA_A_R α_1_/γ_2_ interface, a site recognized as a high-affinity binding pocket for benzodiazepines. Unlike classical benzodiazepines such as diazepam, gidazepam’s hydrazine moiety interacts with hydrophobic amino acids in the binding pocket which disrupts optimal binding stability and weakens gidazepam’s affinity for the GABA_A_R (Mohamad et al., 2023). As a result, gidazepam exhibits high K_i_ values, indicating that it acts as a prodrug at GABA_A_R rather than an active ligand. This mechanism is consistent with pharmacological observations: intoxication with gidazepam typically results in mild anxiolytic effects rather than potent sedative and muscle-relaxant properties observed with traditional benzodiazepines (Azimova and Petelin 2024). In contrast, both desalkylgidazepam and its 3-hydroxy metabolites demonstrate stronger interactions within the receptor pocket, primarily through interactions with polar amino acid residues. Consequently, they display high binding affinities, comparable to diazepam. These findings suggest that desalkylgidazepam and its metabolites are primarily responsible for the pronounced effects associated with gidazepam intoxication.

Furthermore, gidazepam appears to have affinity at additional sites, notably the α_1_β_2_ interface, mainly at the TMD of the A/B interface. Desalkylgidazepam also shows affinity at the β_2_/γ_2_ interface, suggesting additional modes of modulation that may contribute to the pharmacological profiles of these benzodiazepines. In fact, electrophysiological and molecular dynamics studies have shown similar mode of action for diazepam at the TMD of the α_1_β_2_ subunits (Ramerstorfer et al., 2011), as well as high affinity interactions at the β_2_/γ_2_ interface (Wongsamitkul et al., 2017). From a molecular dynamic point of view, these additional binding sites may be less accessible to solvents, and consequently, cyclic compounds such as benzodiazepines may preferentially interact with the α_1_/γ_2_ interface, however, they likely contribute to the overall pharmacodynamic effects and as such should not be ignored (Sigel and Ernst 2018). This clearly depicts the complex benzodiazepine pharmacokinetic and pharmacodynamic interplay and the need for further experimentations. Interestingly, 4-chlorodiazepam, due to the presence of a chlorophenyl modification, lacks compatibility with the GABA_A_R binding pocket. This prevents effective interaction and subsequent receptor activation.

### *In silico* pharmacodynamics on TSPO 18 kDa receptor

Benzodiazepines, including diazepam and 4-chlorodiazepam, interact with the TSPO, stimulating immune-modulation and neurosteroidogenesis. Specifically, these compounds promote the production of allopregnanolone, a neurosteroid known to enhance GABAergic neurotransmission through positive allosteric modulation of GABA_A_R (Wang 2011; Legesse et al., 2023).

The TSPO is a monomeric protein with key binding pockets located within its C-terminal domain, which readily accommodates the benzodiazepines investigated in this study. The high affinity observed for benzodiazepines at TSPO is largely attributable to the presence of adjacent phospholipids, which enhance ligand-receptor interactions via polar group interactions. Our molecular modelling indicates that the stabilization of ligands at TSPO is critically dependent on interactions with Arg43. Remarkably, despite its structural similarity to diazepam and 4-chlorodiazepam, desalkylgidazepam does not exhibit measurable binding affinity for TSPO, suggesting the existence of specific molecular constraints governing ligand recognition.

In contrast, gidazepam demonstrated superior binding efficiency compared to the hydroxylated metabolites of desalkylgidazepam when TSPO was modelled with the POPC phospholipid. However, considering TSPO’s mitochondrial localization, further simulations were conducted with PVCL2, a phospholipid prevalent in mitochondrial membranes. Under these conditions, the binding affinity hierarchy shifted in the order (3*S*)-hydroxy desalkylgidazepam > (3*R*)-hydroxy desalkylgidazepam > gidazepam. This finding suggests that specific lipid environments can modulate benzodiazepine affinity for TSPO, potentially influencing both receptor function and downstream pharmacodynamic activity.

## Conclusion

In this study, we investigated the metabolic transformation of gidazepam and its active metabolite, desalkylgidazepam, by conducting in vitro incubations with pooled human hepatocytes for three hours and using high-resolution mass spectrometry for analysis. Our findings are consistent with *N*-desalkylation as the primary biotransformation pathway for gidazepam, although modifications on gidazepam’s hydrazine chain play a role in the detoxification process. In contrast, desalkylgidazepam appeared to mainly undergo hydroxylations, followed by phase II glucuronidation reactions. Additionally, analysis of an authentic intoxication case indicated the presence of both benzodiazepines. Gidazepam-specific metabolites such as gidazepam-*N*-glucuronide and *N*-acetyl gidazepam were tentatively identified alongside elevated levels of desalkylgidazepam. Notably, we also observed a previously unreported biotransformation pathway for gidazepam, possibly involving deamination and carbonyl reduction of *N*-acetyl gidazepam.

Furthermore, molecular dynamics simulations of GABA_A_R support the hypothesis that gidazepam act as a prodrug, aligning with previous reports. It is also possible that interactions at α_1_β_2_ subunits of GABA_A_R contribute to its anxiolytic effects. Desalkylgidazepam and its 3-hydroxy metabolites exhibited substantial binding interactions at the high-affinity benzodiazepine binding site, with affinities comparable to those of diazepam. Interestingly, desalkylgidazepam showed no apparent activity at the TSPO 18 kDa receptor. However, its (3*S*)- and (3*R*)-hydroxy metabolites demonstrated enhanced potency at the TSPO compared to gidazepam. Taken together, these findings offer valuable insights into the differential pharmacodynamics of benzodiazepines at both the GABA_A_R and TSPO 18 kDa receptor, while highlighting structural features that may influence ligand-receptor interactions across distinct neurobiological targets.

## Supplementary Information

Below is the link to the electronic supplementary material.

Supplementary file1 (DOCX 62 KB)

Supplementary file2 (DOCX 51 KB)

Supplementary file3 (DOCX 53 KB)

Supplementary file4 (DOCX 51 KB)

## Data Availability

Data from this study used in support the current findings are available upon request from the corresponding authors.
