# Supplementary material for: Insights into the human metabolism and in silico receptor activity of gidazepam and desalkylgidazepam
Source: Arch Toxicol. 2025 Dec 8;100(3):959–78. doi: 10.1007/s00204-025-04249-z (PMC12967407; doi:10.1007/s00204-025-04249-z)
Supplement: Supplementary file 1 — Supplementary file1 (DOCX 62 KB) [file 204_2025_4249_MOESM1_ESM.docx]

**Table S1.** Gidazepam and desalkylgidazepam inclusion list for data dependent MS/MS acquisition. Biotransformations designated ^A^ and ^B^ are proposed for gidazepam and desalkylgidazepam, respectively.

| *Transformation* | *Molecular Formula* | *[M^79^Br + H]^+^ [M^79^Br - H]^-^* | *[M^81^Br + H]^+^ [M^81^Br - H]^-^* | *Comments* |
| --- | --- | --- | --- | --- |
| Gidazepam | C_17_H_15_BrN_4_O_2_ | 387.0451  385.0306 | 389.0431  387.0285 | Gidazepam |
| -2C -4H -2N -O | C_15_H_11_BrN_2_O | 315.0127  312.9982 | 317.0107  314.9962 | Desalkylgidazepam |
| +O | C_17_H_15_BrN_4_O_3_ | 403.0400  401.0255 | 405.0380  403.0234 | ^A^Oxidation [hydroxylation] |
| +O | C_15_H_11_BrN_2_O_2_ | 331.0077  328.9931 | 333.0056  330.9911 | ^B^Oxidation [hydroxylation] |
| +6C +8H +7O | C_23_H_23_BrN_4_O_9_ | 579.0721  577.0576 | 581.0701  579.0555 | ^A^Oxidation [hydroxylation] + glucuronidation |
| +2C +2H +O | C_19_H_17_BrN_4_O_3_ | 429.0557  427.0411 | 431.0536  429.0391 | ^A^*N*-Acetylation |
| +2O | C_17_H_15_BrN_4_O_4_ | 419.0349  417.0204 | 421.0329  419.0183 | ^A^Oxidation [double hydroxylation] |
| +6C +9H +7O | C_21_H_19_BrN_2_O_8_ | 507.0397  505.0252 | 509.0377  507.0232 | ^B^Oxidation [hydroxylation] + glucuronidation |
| +2H | C_17_H_17_BrN_4_O_2_ | 389.0608  387.0462 | 391.0587  389.0442 | ^A^Reduction |
| +2H +O | C_17_H_17_BrN_4_O_3_ | 405.0557  403.0411 | 407.0536  405.0391 | ^A^Oxidation [hydroxylation] + reduction |
| +6C +8H +6O | C_23_H_23_BrN_4_O_8_ | 563.0772  561.0626 | 565.0752  563.0606 | ^A^*N*-Glucuronidation |
| +6C +8H +8O | C_23_H_23_BrN_4_O_10_ | 595.0670  593.0525 | 597.0650  595.0504 | ^A^Oxidation [double hydroxylation] + glucuronidation |
| +2O | C_15_H_11_BrN_2_O_3_ | 347.0026  344.9880 | 349.0005  346.9860 | ^B^Oxidation [double hydroxylation] |
| +2C +2H +2O | C_19_H_17_BrN_4_O_4_ | 445.0506  443.0360 | 447.0486  445.0340 | ^A^*N*-Acetylation + oxidation [hydroxylation] |
| +6C +9H +8O | C_21_H_19_BrN_2_O_9_ | 523.0347  521.0201 | 525.0326  523.0181 | ^B^Oxidation [double hydroxylation] + glucuronidation |
| +2C +3O | C_19_H_15_BrN_4_O_5_ | 459.0299  457.0153 | 461.0278  459.0133 | ^A^*N*-Acetylation + carboxylation |
| +2H +2O | C_17_H_17_BrN_4_O_4_ | 421.0506  419.0360 | 423.0486  421.0340 | ^A^Oxidation [double hydroxylation] + reduction/ dihydrodiol formation |
| -2H -2N +O | C_17_H_13_BrN_2_O_3_ | 373.0182  371.0037 | 375.0162  373.0016 | ^A^Hydrazide reduction to carboxylic acid |
| +4O +S | C_17_H_15_BrN_4_O_6_S | 482.9968  480.9823 | 484.9948  482.9802 | ^A^Oxidation [hydroxylation] + sulfation |
| +6C +10H +6O | C_23_H_25_BrN_4_O_8_ | 565.0928  563.0783 | 567.0908  565.0763 | ^A^Reduction + glucuronidation |
| +2H | C_15_H_13_BrN_2_O | 317.0284  315.0138 | 319.0264  317.0118 | ^B^Reduction |
| +2H +O | C_15_H_13_BrN_2_O_2_ | 333.0233  331.0088 | 335.0213  333.0067 | ^B^Reduction + oxidation [hydroxylation] |
| -2H | C_17_H_13_BrN_4_O_2_ | 385.0295  383.0149 | 387.0274  385.0129 | ^A^Formation of C-C double bond |
| +6C +10H +7O | C_23_H_25_BrN_4_O_9_ | 581.0878  579.0732 | 583.0857  581.0712 | ^A^Oxidation [hydroxylation] + reduction + glucuronidation |
| +6C +9H +6O | C_21_H_19_BrN_2_O_7_^+^ | 490.0370  - | 492.0350  - | ^B^*N*-Glucuronidation |
| +5O +S | C_17_H_15_BrN_4_O_7_S | 498.9918  496.9772 | 500.9897  498.9752 | ^A^Oxidation [double hydroxylation] + sulfation |
| +4O +S | C_15_H_11_BrN_2_O_5_S | 410.9645  408.9499 | 412.9624  410.9479 | ^B^Oxidation [hydroxylation] + sulfation |
| +6C +9H +7O | C_21_H_21_BrN_2_O_8_ | 509.0554  507.0408 | 511.0534  509.0388 | ^B^Oxidation [hydroxylation] + glucuronidation + reduction |
| +2H +2O | C_15_H_13_BrN_2_O_3_ | 349.0182  347.0036 | 351.0162  349.0016 | ^B^Oxidation [double hydroxylation] + reduction/ dihydrodiol formation |
| C +2H +O | C_18_H_17_BrN_4_O_3_ | 417.0557  415.0411 | 419.0536  417.0391 | ^A^Oxidation [hydroxylation] + methylation |
| C +2H +2O | C_18_H_17_BrN_4_O_4_ | 433.0506  431.0360 | 435.0486  433.0340 | ^A^Oxidation [double hydroxylation] + methylation |
| +2H +4O +S | C_15_H_13_BrN_2_O_5_S | 412.9801  410.9656 | 414.9781  412.9635 | ^B^Oxidation [hydroxylation] + sulfation + reduction |
| +C +2H +O | C_16_H_13_BrN_2_O_2_ | 345.0233  343.0088 | 347.0213  345.0067 | ^B^Oxidation [hydroxylation] + methylation |
| +C +2H +2O | C_16_H_13_BrN_2_O_3_ | 361.0182  359.0037 | 363.0162  361.0016 | ^B^Oxidation [double hydroxylation] + methylation |
| -2H | C_15_H_9_BrN_2_O | 312.9971  310.9825 | 314.9951  312.9805 | ^B^Desaturation |
| +6C +11H +6O | C_21_H_21_BrN_2_O_7_ | 493.0605  491.0459 | 495.0584  493.0439 | ^B^Reduction + glucuronidation |
| +5O +S | C_15_H_11_BrN_2_O_6_S | 426.9594  424.9448 | 428.9574  426.9428 | ^B^Oxidation [Double hydroxylation] + sulfation |
| -Br | C_17_H_16_N_4_O_2_ | 309.1346  307.1200 | -  - | ^A^Debromination |
| -Br | C_15_H_11_N_2_O | 237.1022  235.0877 | -  - | ^B^Debromination |
| -Br + O | C_17_H_16_N_4_O_3_ | 325.1295  323.1150 | -  - | ^A^Debromination + oxidation [hydroxylation] |
| -Br +O | C_15_H_11_N_2_O_2_ | 253.0972  251.0826 | -  - | ^B^Debromination + hydroxylation |
| +10C +16H +3N +6O +S -Br | C_27_H_31_N_7_O_8_S | 614.2028  612.1882 | -  - | ^A^Glutathione conjugation [on Br] |
| +10C +16H +3N +6O +S -Br | C_25_H_27_BrN_5_O_7_S | 542.1704  540.1558 | -  - | ^B^Glutathione conjugation |
| +10C +14H +2N +5O +S | C_27_H_29_BrN_6_O_8_S | 677.1024  675.0878 | 679.1003  677.0858 | ^A^Glutathione conjugation [on NH2] |
