# Supplementary material for: Insights into the human metabolism and in silico receptor activity of gidazepam and desalkylgidazepam
Source: Arch Toxicol. 2025 Dec 8;100(3):959–78. doi: 10.1007/s00204-025-04249-z (PMC12967407; doi:10.1007/s00204-025-04249-z)
Supplement: Supplementary file 2 — Supplementary file2 (DOCX 51 KB) [file 204_2025_4249_MOESM2_ESM.docx]

**Table S2.** Transformations for generating potential phase I and phase II metabolites of gidazepam and desalkylgidazepam in Compound Discoverer data mining software

| Phase I | Dehydration (H2 O → )  Desaturation (H2 → ) Dihydrodiol formation ( → H2 O2)  Hydration ( → H2 O)  Ketone formation (O → H2) Nitro reduction (O2 → H2)  Oxidation ( → O) Oxidative deamination to alcohol (H2 N → HO)  Oxidative deamination to ketone (H3 N → HO) Oxidative debromination (Br → HO) Reduction ( → H2) Reduction debromination (Br → H) |
| --- | --- |
| Phase II | Acetylation ( H → C2 H3 O) Cysteine conjugation on Br (Br → C3 H6 N O2 S) Cysteine conjugation on nitrile ( → C3 H6 N O2 S) Cysteine-glycine conjugation on Br (Br → C5 H9 N2 O3 S) Cysteine-glycine conjugation on nitrile ( → C5 H9 N2 O3 S) Glucuronide conjugation (H → C6 H9 O6) Glycine conjugation at NH_2_ (H → C2 H3 O2)  GSH conjugation on Br (Br → C10 H16 N3 O6 S) GSH conjugation on nitrile ( → C10 H16 N3 O6 S)  Methylation ( H → C H3) Sulfation (H → H O3 S) Thiol conjugation (H2 S → ) |
| Others | Desalkylgidazepam *N*-glucuronide conjugation ( → C6 H9 O6) Gidazepam *N*-dealkylation (C5 H5 N2 O → H)  Gidazepam *N*-glucuronide conjugation ( → C6 H9 O6)  Gidazepam hydrazide reduction (H3 N2 → H O)  Gidazepam hydrazide reduction (H3 N2 → H O) |
| Max # Dealkylation | 2 |
| Max # Phase II | 2 |
| Max # of reactions | 5 |
