# Supplementary material for: Insights into the human metabolism and in silico receptor activity of gidazepam and desalkylgidazepam
Source: Arch Toxicol. 2025 Dec 8;100(3):959–78. doi: 10.1007/s00204-025-04249-z (PMC12967407; doi:10.1007/s00204-025-04249-z)
Supplement: Supplementary file 3 — Supplementary file3 (DOCX 53 KB) [file 204_2025_4249_MOESM3_ESM.docx]

**Table S3.** Biotransformation, elemental structure, probability score and Simplified Molecular-Input Line Entry Specification (SMILES) of gidazepam metabolites predicted using GLORYx free-base webtool.

| *ID* | *Transformation* | *Structure* | *Score (%)* | *SMILES* |
| --- | --- | --- | --- | --- |
| Parent | Gidazepam | C_17_H_15_BrN_4_O_2_ | - | O=C1CN=C(c2cc(Br)ccc2N1CC(=O)NN)c1ccccc1 |
| pM1 | *N*-Acetylation | C_19_H_17_BrN_4_O_3_ | 80 | O=C1CN=C(c2cc(Br)ccc2N1CC(=O)NNC(=O)C)c1ccccc1 |
| pM1-1 | + Hydroxylation | C_19_H_17_BrN_4_O_4_ | 31 | ON(NC(=O)C)C(=O)CN1C(=O)CN=C(c2cc(Br)ccc12)c1ccccc1 |
| pM1-2 | + Carboxylation | C_19_H_15_BrN_4_O_5_ | 31 | OC(=O)C(=O)NNC(=O)CN1C(=O)CN=C(c2cc(Br)ccc12)c1ccccc1 |
| pM1-3 | + De-acetylation | C_17_H_15_BrN_4_O_2_ | 31 | O=C1CN=C(c2cc(Br)ccc2N1CC(=O)NN)c1ccccc1 |
| pM1-4 | + Hydroxylation | C_19_H_17_BrN_4_O_4_ | 31 | O=C1CN=C(c2cc(Br)ccc2N1CC(=O)NNC(=O)CO)c1ccccc1 |
| pM2 | Hydroxylation [phenyl] | C_17_H_15_BrN_4_O_3_ | 31 | O=C1CN=C(c2cc(Br)ccc2N1CC(=O)NN)c1cc(O)ccc1 |
| pM2-1 | + *O*-Sulfation | C_17_H_15_BrN_4_O_6_S | 30 | O=S(O)(=O)Oc1cc(ccc1)C1=NCC(=O)N(CC(=O)NN)c2ccc(Br)cc12 |
| pM2-2 | + *O*-Glucuronidation | C_22_H_23_BrN_4_O_8_ | 29 | OC1OC(Oc2cc(ccc2)C2=NCC(=O)N(CC(=O)NN)c3ccc(Br)cc23)C(O)C(O)C1O |
| pM2-3 | + *O*-Methylation | C_18_H_17_BrN_4_O_3_ | 29 | O=C1CN=C(c2cc(Br)ccc2N1CC(=O)NN)c1cc(ccc1)OC |
| pM2-4 | + *N-*Acetylation | C_19_H_17_BrN_4_O_4_ | 25 | O=C1CN=C(c2cc(Br)ccc2N1CC(=O)NNC(=O)C)c1cc(O)ccc1 |
| pM3 | Hydroxylation + methoxylation | C_18_H_17_BrN_4_O_4_ | 31 | O=C1CN=C(c2cc(Br)ccc2N1CC(=O)NN)c1cc(cc(O)c1)OC |
| pM3-1 | + *N-*Acetylation | C_20_H_19_BrN_4_O_5_ | 25 | O=C1CN=C(c2cc(Br)ccc2N1CC(=O)NNC(=O)C)c1cc(cc(O)c1)OC |
| pM3-2 | + *O*-Glucuronidation | C_23_H_25_BrN_4_O_9_ | 25 | OC1OC(Oc2cc(cc(c2)C2=NCC(=O)N(CC(=O)NN)c3ccc(Br)cc23)OC)C(O)C(O)C1O |
| pM4 | Hydroxylation | C_17_H_15_BrN_4_O_3_ | 31 | O=C1CN=C(c2cc(Br)ccc2N1CC(=O)NN)c1ccc(O)cc1 |
| pM5 | Hydroxylation | C_17_H_15_BrN_4_O_3_ | 28 | ON(N)C(=O)CN1C(=O)CN=C(c2cc(Br)ccc12)c1ccccc1 |
| pM6 | Hydrazide reduction + carboxylation | C_17_H_13_BrN_2_O_3_ | 28 | OC(=O)CN1C(=O)CN=C(c2cc(Br)ccc12)c1ccccc1 |
