# Supplementary material for: Insights into the human metabolism and in silico receptor activity of gidazepam and desalkylgidazepam
Source: Arch Toxicol. 2025 Dec 8;100(3):959–78. doi: 10.1007/s00204-025-04249-z (PMC12967407; doi:10.1007/s00204-025-04249-z)
Supplement: Supplementary file 4 — Supplementary file4 (DOCX 51 KB) [file 204_2025_4249_MOESM4_ESM.docx]

**Table S4.** Biotransformation, elemental structure, probability score and Simplified Molecular-Input Line Entry Specification (SMILES) of desalkylgidazepam metabolites predicted using GLORYx free-base webtool.

| *ID* | *Transformation* | *Structure* | *Score (%)* | *SMILES* |
| --- | --- | --- | --- | --- |
| Parent | Desalkylgidazepam | C_15_H_11_BrN_2_O | - | O=C1CN=C(c2cc(Br)ccc2N1)c1ccccc1 |
| pM1 | Hydroxylation + *O*-methylation | C_16_H_13_BrN_2_O_3_ | 32 | O=C1CN=C(c2cc(OC)c(O)cc2)c2cc(Br)ccc2N1 |
| pM1-1 | + *O*-Glucuronidation | C_21_H_21_BrN_2_O_8_ | 37 | OC1OC(Oc2ccc(cc2OC)C2=NCC(=O)Nc3ccc(Br)  cc23)C(O)C(O)C1O |
| pM2 | Hydroxylation | C_15_H_11_BrN_2_O_2_ | 32 | O=C1CN=C(c2ccc(O)cc2)c2cc(Br)ccc2N1 |
| pM2-1 | + *O*-Glucuronidation | C_20_H_19_BrN_2_O_7_ | 31 | OC1OC(Oc2ccc(cc2)C2=NCC(=O)Nc3ccc(Br)  cc23)C(O)C(O)C1O |
| pM2-2 | + *O*-Sulfation | C_15_H_11_BrN_2_O_5_S | 30 | O=S(O)(=O)Oc1ccc(cc1)C1=NCC(=O)Nc2ccc(Br)cc12 |
| pM2-3 | + *O*-Methylation | C_16_H_13_BrN_2_O_2_ | 30 | O=C1CN=C(c2cc(Br)ccc2N1)c1ccc(cc1)OC |
| pM3 | Hydroxylation | C_15_H_11_BrN_2_O_2_ | 23 | O=C1CN=C(c2cc(O)ccc2)c2cc(Br)ccc2N1 |
